# Interactive Case-Based Childhood Adversity and Trauma-Informed Care Electronic Modules for Pediatric Primary Care

**DOI:** 10.15766/mep_2374-8265.10990

**Published:** 2020-10-12

**Authors:** Binny Chokshi, Kuan-Lung Daniel Chen, Lee Beers

**Affiliations:** 1 General Pediatrician, Children's National Health System; Assistant Professor, Department of Pediatrics, George Washington University School of Medicine and Health Sciences; 2 Senior Researcher, Building Community Resilience, Sumner M. Redstone Global Center for Prevention and Wellness, Milken Institute School of Public Health, George Washington University; 3 General Pediatrician, Children's National Health System; Medical Director of Community Health and Advocacy, Child Health Advocacy Institute; Associate Professor, Department of Pediatrics, George Washington University School of Medicine and Health Sciences

**Keywords:** Adverse Childhood Experiences, Trauma-Informed Care, Resiliency, Primary Care, Pediatrics, Online/Distance Learning, Self-Regulated Learning, Virtual Learning

## Abstract

**Introduction:**

Training health professionals for the skills and capacity to respond adequately to children and adults who have been exposed to adverse childhood experiences is recognized as an essential need in health care. Accessible opportunities to educate physicians and physician-trainees are limited.

**Methods:**

Four computer-based e-modules were created focusing on addressing childhood adversity and implementing trauma-informed care in the pediatric primary care setting. These childhood adversity and trauma-informed care (CA-TIC) e-modules were designed as an individualized, self-directed experience to allow for distance learning with flexibility to be embedded into existing coursework. To foster an engaging learning environment, we narrated the modules, prioritized images, and included the opportunity for participant interaction via multiple-choice and short-answer questions. Twenty-eight pediatric residents, two medical students, four attending physicians, and one fellow at Children's National Hospital completed the e-modules.

**Results:**

Overall, participants rated the CA-TIC e-modules 4.6 (*SD* = 0.5) out of 5 for design and quality. Using paired *t* tests and Wilcoxon signed rank tests, we found statistically significant score increases from presession to postsession for participants' knowledge, attitudes, practice, and confidence related to CA-TIC. The most commonly cited learning points and practice changes included asking about trauma in practice and the seven C's of resilience.

**Discussion:**

A trauma-informed, strengths-based approach to care can assist health care providers in mitigating the link between adversity and related poor health outcomes. The CA-TIC e-modules provide an opportunity to train health professionals using an innovative, self-directed, and low-resource mechanism.

## Educational Objectives

By the end of this activity, learners will be able to:

1. Describe how childhood trauma and adversity can affect health outcomes.
2. List key principles of trauma-informed care and pediatric resiliency building.
3. Describe how to informally and formally screen for and identify a history of trauma and adversity during pediatric patient encounters.
4. Apply trauma-informed care principles, including resiliency building, in daily patient interactions.

## Introduction

A seminal study by Kaiser Permanente and the Centers for Disease Control and Prevention in 1998 examined 10 specific types of childhood trauma (such as exposure to physical, sexual, and verbal abuse or household dysfunction), now known as adverse childhood experiences (ACEs), and showed a direct, dose-dependent relationship between ACEs and poor adult health.^1^ Subsequent studies have shown that the mediating factor in the association between ACEs and poor health outcomes is high levels of toxic stress.^2^ Exposure to childhood adversity can lead to an intense, frequent, and sustained activation of the body's stress response, and over time, this can result in unhealthy behaviors, chronic health conditions, and even early death.^2^

Given the link to adult chronic disease, research has highlighted the health care costs of not addressing childhood traumatic exposures,^3^ and addressing childhood adversity has increasingly become a priority across sectors such as nursing, medicine, and social work.^4^ Training to strengthen health professionals' skills and capacity to respond adequately to children and adults who have been exposed to ACEs has been recognized as an essential need in health care.^1^ Health care providers are uniquely positioned to mediate the link between traumatic exposures and poor health. Increasing the ability of health care providers to recognize and respond to ACEs by using a trauma-informed approach to care can not only buffer the long-term negative physical and mental health impacts of traumatic exposures^5^ but also improve patient care indicators, such as number of physician office and emergency room visits.^6^ Discussion of a past history of traumatic exposures can also increase patient-centered medical care, which in turn can improve health outcomes.^7^

Despite these benefits, health care practitioners have reported discomfort in discussing trauma with their patients,^8^ and the impact of traumatic exposure is not routinely addressed in our general health care systems.^9^ To effectively address the effects of ACEs, health care providers must not only understand the impact on an individual's health and well-being but also learn the skills to employ a trauma-informed approach to care, which acknowledges the events, experiences, and effects of individual trauma.^10,11^ Current opportunities and models to educate physicians and physician-trainees in the science of ACEs and the ideals of a trauma-informed approach to care are limited.^7,9^

A recent scoping review of the current state of trauma-informed education embedded in health sciences curricula identified 22 articles that described trauma-informed educational curricula.^12^ Of these, only two (9%) were in nonpsychiatric medicine; most (68%) were in social work and psychology.^12^ One of the medical studies described 6 hours of ACEs science and trauma-informed care (TIC) training for medical students at the University of California, Davis, which included three 2-hour modules, with lectures complemented by experiential learning.^5^ A posttraining questionnaire revealed that students felt the training increased their ability to recognize clinical manifestations of ACE exposure in patients and their knowledge regarding asking about and responding to patient ACE disclosures.^5^ The second medical study delivered an ACE and TIC curriculum to 967 students from nine health profession education programs, ranging from medicine to optometry.^13^ Postcurricular survey responses demonstrated an increase in understanding of ACEs and TIC.^13^ Additional examples beyond the scope of this review include a TIC training focused on communication for primary care physicians,^7^ training in TIC for pediatric residents using the lens of caring for substance-exposed mother-infant dyads,^14^ and a 1-hour TIC training across a pediatric health care network.^15^

*MedEdPORTAL* has published only two resources related to ACEs. One describes a mandatory 3-hour workshop for first-year medical students at Rutgers New Jersey School of Medicine including didactics combined with small-group discussion,^16^ and the second reports on a 25-minute self-directed module for pediatric residents embedded into an advocacy rotation.^17^ *MedEdPORTAL* has also published two resources related to TIC, one on caring for female sexual assault survivors^18^ and the second on trauma-informed physical examination techniques.^19^ With the exception of the 25-minute self-directed module,^18^ these learning opportunities require faculty in place to deliver the content. While this in-person element allows for rich discussion and active learning, limitations include faculty availability, scheduling, and finding protected time in existing curricula.

We designed our e-modules in response to this need. Our interactive case-based childhood adversity and trauma-informed care (CA-TIC) e-modules for pediatric primary care allow ACE and TIC education to be delivered via distance learning, are available to multiple audiences with flexibility, and can easily be embedded into existing educational rotations and coursework. The CA-TIC e-modules are designed to be an individual case-based 2-hour educational experience. There is a voice-over for the entirety of the CA-TIC e-modules, which simulates in-person education. To foster an active learning environment, the e-modules include an opportunity for participant interaction via multiple-choice and short-answer questions. The overarching goal of the CA-TIC e-modules is to advance pediatric practice and child health through the provision of interactive didactics to increase knowledge, skills, attitudes, and confidence regarding trauma-informed approaches and resiliency building in daily clinical practice. The target audience of the CA-TIC e-modules is practitioners engaged in pediatric primary care, ranging from medical students to attending physicians.

## Methods

The CA-TIC e-modules have four components: a premodule and three case-based e-modules (Appendices A–D). The titles and objectives of the individual e-modules are listed in Table 1. The first author, Dr. Binny Chokshi, developed the content for the e-modules after extensive review of material from the Substance Abuse and Mental Health Services Administration (SAMHSA) related to a trauma-informed approach^11^ and then adapted the content to be relevant for a medical audience. She then reviewed Ginsburg's seven C's of resilience model^20^ and incorporated it with SAMHSA's trauma-informed principles to create a resiliency-focused, trauma-informed approach to pediatric primary care. Lastly, she reviewed the American Academy of Pediatrics' recommendations on addressing childhood adversity in pediatric primary care to develop the content for the last e-module.^21,22^ Citations for images utilized in the CA-TIC e-modules can be found in Appendix E.

**Table 1.**
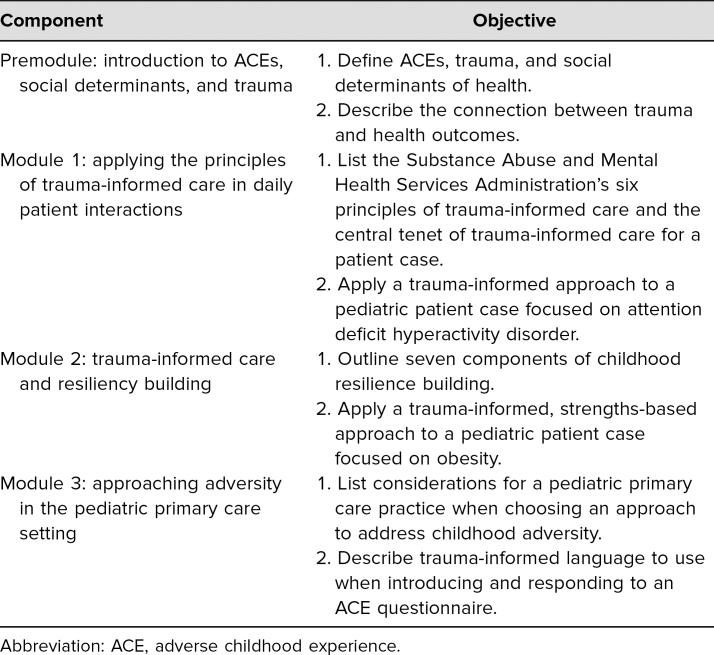
Childhood Adversity and Trauma-Informed Care E-module Components and Objectives

After developing the content, Dr. Chokshi worked closely with the instructional design team at Children's National Hospital (CNH) to translate it into a format suitable for successful e-module education, relying on Mayer's nine research-based principles for instructional design of multimedia lessons.^23^ To reduce extraneous processing,^23^ the team took care to limit text on the screen, use bullets and timed text when appropriate, and link on-screen text with corresponding graphics. To manage essential processing,^23^ the content was separated into three distinct e-modules, preceded by a premodule featuring important definitions and a brief overview of the relevant science. In addition, the team designed the CA-TIC e-modules to require the user to interact with the interface through short-answer questions and use of a Next button to advance through the e-modules. The team also prioritized delivery of learning points via narration over written on-screen text and encouraged the use of images to correspond to the narration (vs. on-screen text). Lastly, to foster generative processing,^23^ the team created an on-screen avatar that resembled Dr. Chokshi, who also narrated a majority of the e-modules.

We incorporated the CA-TIC e-modules into a 1-week advocacy rotation required for second-year categorical pediatric residents at CNH. This rotation gave a brief overview of five social determinants of health, with each day dedicated to an educational experience related to a specific social determinant of health. Residents were assigned the CA-TIC e-modules on the day focused on ACEs and were given protected time for e-module completion. Because the CA-TIC e-modules were intended to be an introduction to ACEs and TIC, there were no prerequisite knowledge requirements. The e-modules were also offered as a voluntary activity to medical students doing elective rotations with the Healthy Generations Teen-Tot Program through CNH, to general academic pediatric fellows, and to primary care attending physicians at CNH.

We evaluated the impact of the course by utilizing a presession and postsession questionnaire (Appendix F). We developed a 17-item questionnaire with four categories—knowledge (five items), attitudes (five items), practice (four items), and confidence (three items)—all scored on 5-point Likert scales. The postsession questionnaire also included one item to rate the CA-TIC e-modules overall on a 5-point Likert scale and three short-answer questions related to course-specific feedback. The pre-postsession evaluation was embedded into the CA-TIC e-module interface, facilitating ease of completion.

We conducted statistical tests using the Stata 15 software. Pre- and postsession scores for each survey item were compared using a paired *t* test if presession and postsession scores each showed a normal distribution. If one or both of the scores had a nonnormal distribution, a Wilcoxon signed rank test was performed. Distribution normality was assessed using the Doornik-Hansen multivariate normality test. The CNH Institutional Review Board reviewed the CA-TIC evaluation protocol and deemed it to be exempt.

## Results

A total of 35 participants completed the CA-TIC e-modules and the presession and postsession questionnaires. Among them, 28 were resident physicians (80%), four were attending physicians (11%), two were medical students (6%), and one was a fellow (3%). Thirty-one participants (89%) reported practicing in academic centers, two (6%) in private clinics, and two (6%) in other practice settings.

As shown in Table 2, our assessments found a statistically significant score increase from presession to postsession for each survey item. The largest magnitude of change was seen in items related to knowledge, with questions related to the central tenet of TIC, the six principles of TIC, and the components of childhood resilience each yielding a mean score increase of more than 2 points. This was likely due to a low starting mean score for the three items, as they received the three lowest presession scores. When averaging the scores across knowledge items, knowledge as a category had a statistically significant increase of 1.9 points.

**Table 2.**
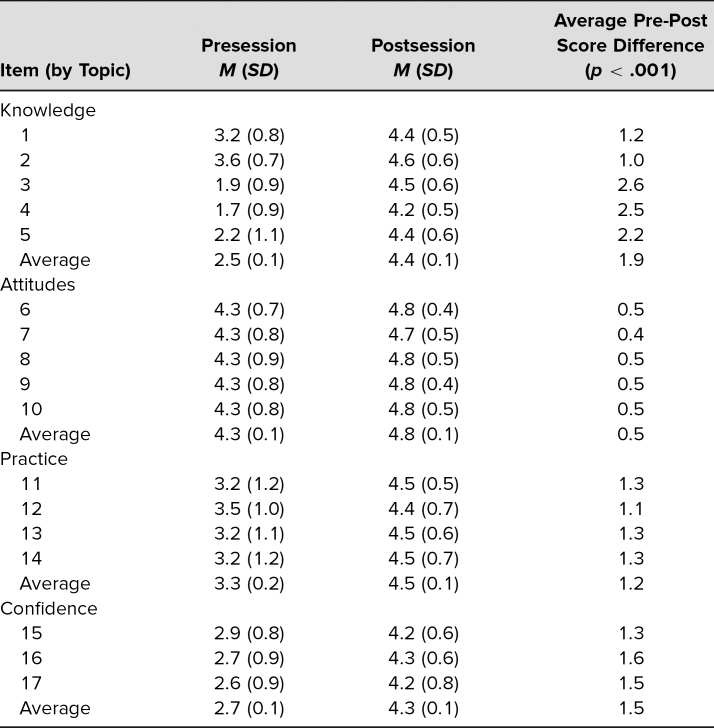
Childhood Adversity and Trauma-Informed Care E-modules Evaluation Results

The smallest changes were seen in attitude items, all of which increased by less than 1 point from presession to postsession. This result was likely due to the high presession mean scores, all of which were greater than 4, allowing attitude scores minimal room for increase. The average presession attitude score was 4.3 out of 5, which increased to 4.8 after the session. Despite the smaller change, the postsession increases for each attitude item, as well as for attitudes as a category, were statistically significant.

As shown in Table 2, the presession to postsession increases for each practice and confidence item, as well as for practice and confidence as categories, were also statistically significant. All of the postsession mean scores were 4 points or higher, suggesting the course not only increased participant scores but consistently helped participants reach a high level of knowledge, attitudes, likelihood to practice TIC, and confidence at providing TIC.

In the postsession questionnaire, participants were asked to provide qualitative comments and reflect on two learning points they took away from the course. The most common responses concerned the seven C's of resilience and information related to screening for trauma—including why screening is important and how to screen, each mentioned by 10 participants (29%). When asked about the two practice changes they would make as a result of the course, 12 participants (34%) mentioned screening for trauma, and five participants (14%) mentioned actively applying the seven C's to build resilience.

**Figure 1.**
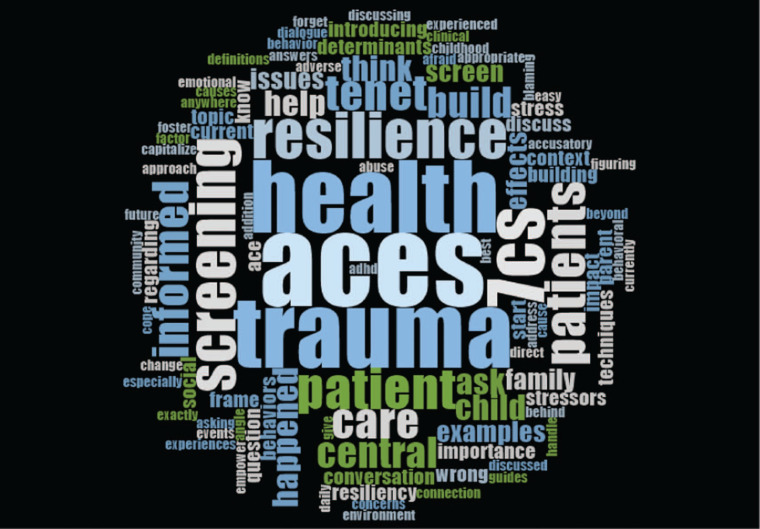
Word cloud: “Please list two specific learning points that you will take away from this course.”

**Figure 2.**
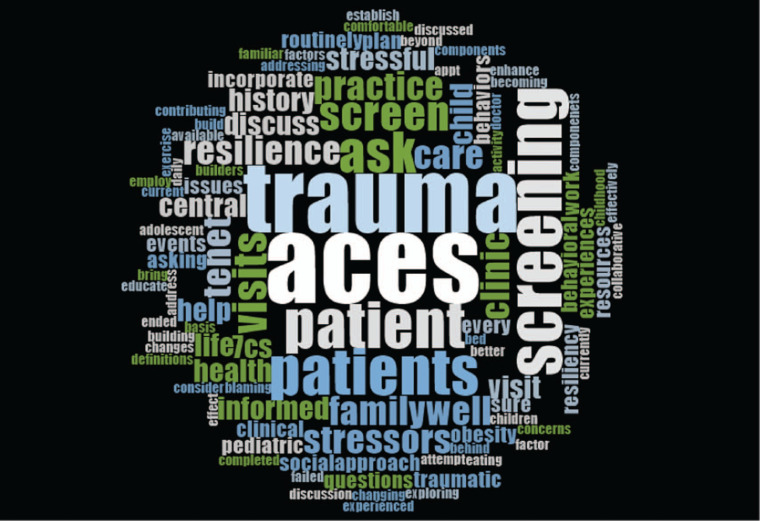
Word cloud: “Please list two specific changes that you plan to make in your clinical practice as a result of this course.”

We created word clouds using free-text responses to the two questions. As shown in Figure 1, the most common words for takeaway learning points were ACEs, trauma, health, resilience, and screen/screening. As shown in Figure 2, the most commonly used words to describe changes participants would make to their clinical practice included trauma, ACEs, and screening, along with the verbs of ask, screen, care, discuss, help. Together, these key words suggest that the CA-TIC e-modules facilitate active application of knowledge and align with the overall CA-TIC e-module objectives.

## Discussion

Providing health professionals with training on responding to children and adults who have been exposed to ACEs is essential, but accessible opportunities to educate physicians and physician-trainees have been limited. Our innovative CA-TIC e-modules met this educational need and increased participant knowledge, attitudes, practice, and confidence related to ACEs and TIC.

Our CA-TIC e-modules provide an opportunity to train pediatric practitioners using an individualized, flexible, and low-resource mechanism. The e-modules can be easily accessed and therefore can be used as distance learning or embedded into an existing curriculum or rotation experience. Another benefit of the CA-TIC e-modules is that they are applicable across the spectrum of medical professionals, ranging from medical students to attending physicians.

Embedding the CA-TIC e-modules into an existing rotation for pediatric residents at CNH and protecting residents' time to complete the activity were essential for e-module completion. Without protected time, continuing medical education credit, or additional division-sponsored incentives, it has been difficult to involve pediatric primary care attending physicians at CNH. To address this challenge, we have begun discussions with hospital leadership regarding how best to promote educational opportunities in alignment with the institution's strategic plan.

One limitation is that the postsession evaluation was done immediately following participation in the CA-TIC e-modules. Future directions include 3- to 6-month follow-up to assess for behavior change in clinical practice. In addition, our study sample mostly comprised pediatric residents from one academic institution. Limitations of the content of the CA-TIC e-modules include lack of inclusion of evidence-based opportunities for primary prevention of ACEs and the fact that all cases in the CA-TIC e-modules are pediatric focused.

To expand the evaluation and reach of the CA-TIC e-modules, we plan to disseminate them to the wider Washington, DC, community pediatrician network and beyond. We also recognize the importance of content regarding wellness opportunities for providers who are practicing TIC. We are currently working as part of a multidisciplinary team that includes mental health professionals to finalize an interactive e-module on promoting provider well-being that focuses on compassion fatigue, vicarious trauma, and burnout, to complement the CA-TIC e-modules.

A trauma-informed, strengths-based approach to care can assist health care providers in mitigating the link between adversity and related poor health outcomes. Our CA-TIC e-modules contribute to the growing educational compendium related to ACEs and TIC and provide an opportunity to train a workforce of health professionals to address childhood adversity.

## Appendices

CA-TIC Premodule folderCA-TIC Module 1 folderCA-TIC Module 2 folderCA-TIC Module 3 folderImage Citations.docxCA-TIC Evaluation.docx
All appendices are peer reviewed as integral parts of the Original Publication.
