# Supplementary material for: Interactive Case-Based Childhood Adversity and Trauma-Informed Care Electronic Modules for Pediatric Primary Care
Source: MedEdPORTAL. 2020 Oct 12;16:10990. doi: 10.15766/mep_2374-8265.10990 (PMC7549390; doi:10.15766/mep_2374-8265.10990)
Supplement: Supplementary file 1 — CA-TIC Premodule folderCA-TIC Module 1 folderCA-TIC Module 2 folderCA-TIC Module 3 folderImage Citations.docxCA-TIC Evaluation.docx [file mep_2374-8265.10990-s001.zip › D. CA-TIC Module 3/index.html]

Trauma Informed Care - Module 3


Trauma Informed Care - Module 3
