# Supplementary figures and images for: Interactive Case-Based Childhood Adversity and Trauma-Informed Care Electronic Modules for Pediatric Primary Care
Source: MedEdPORTAL. 2020 Oct 12;16:10990. doi: 10.15766/mep_2374-8265.10990 (PMC7549390; doi:10.15766/mep_2374-8265.10990)

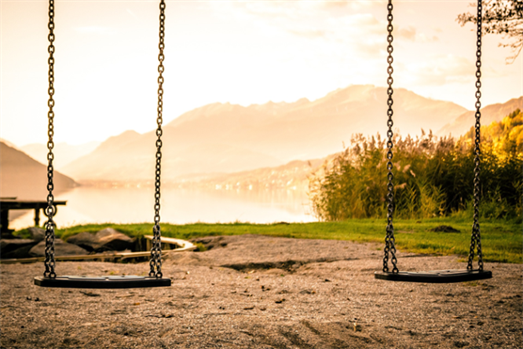

Supplement: Supplementary file 1 — CA-TIC Premodule folderCA-TIC Module 1 folderCA-TIC Module 2 folderCA-TIC Module 3 folderImage Citations.docxCA-TIC Evaluation.docx [file mep_2374-8265.10990-s001.zip › A. CA-TIC Premodule/mobile/5afApGtcOBT_DX666_DY666_CX524_CY349.png]

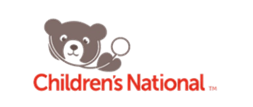

Supplement: Supplementary file 1 — CA-TIC Premodule folderCA-TIC Module 1 folderCA-TIC Module 2 folderCA-TIC Module 3 folderImage Citations.docxCA-TIC Evaluation.docx [file mep_2374-8265.10990-s001.zip › A. CA-TIC Premodule/mobile/5glQ9yBrUJz_DX322_DY322_CX253_CY108.png]

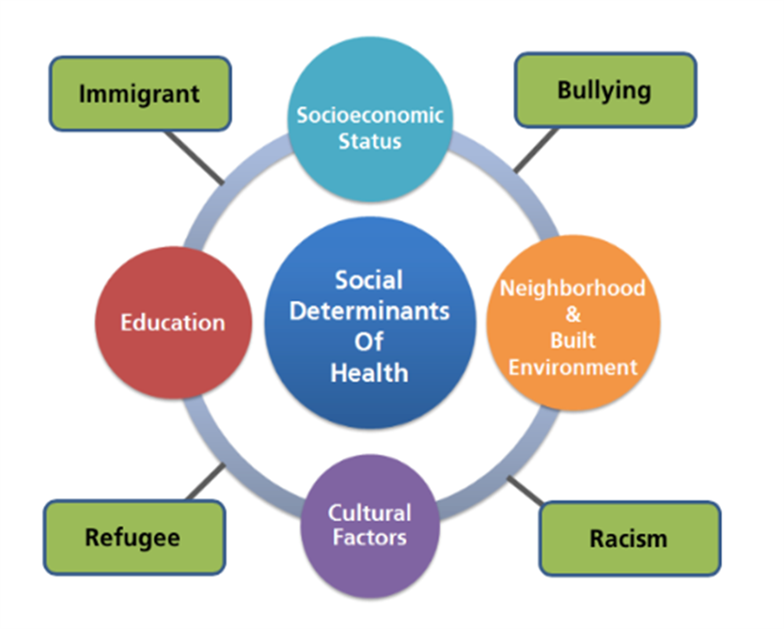

Supplement: Supplementary file 1 — CA-TIC Premodule folderCA-TIC Module 1 folderCA-TIC Module 2 folderCA-TIC Module 3 folderImage Citations.docxCA-TIC Evaluation.docx [file mep_2374-8265.10990-s001.zip › A. CA-TIC Premodule/mobile/5oxTducMFSw_DX998_DY998_CX785_CY629.png]

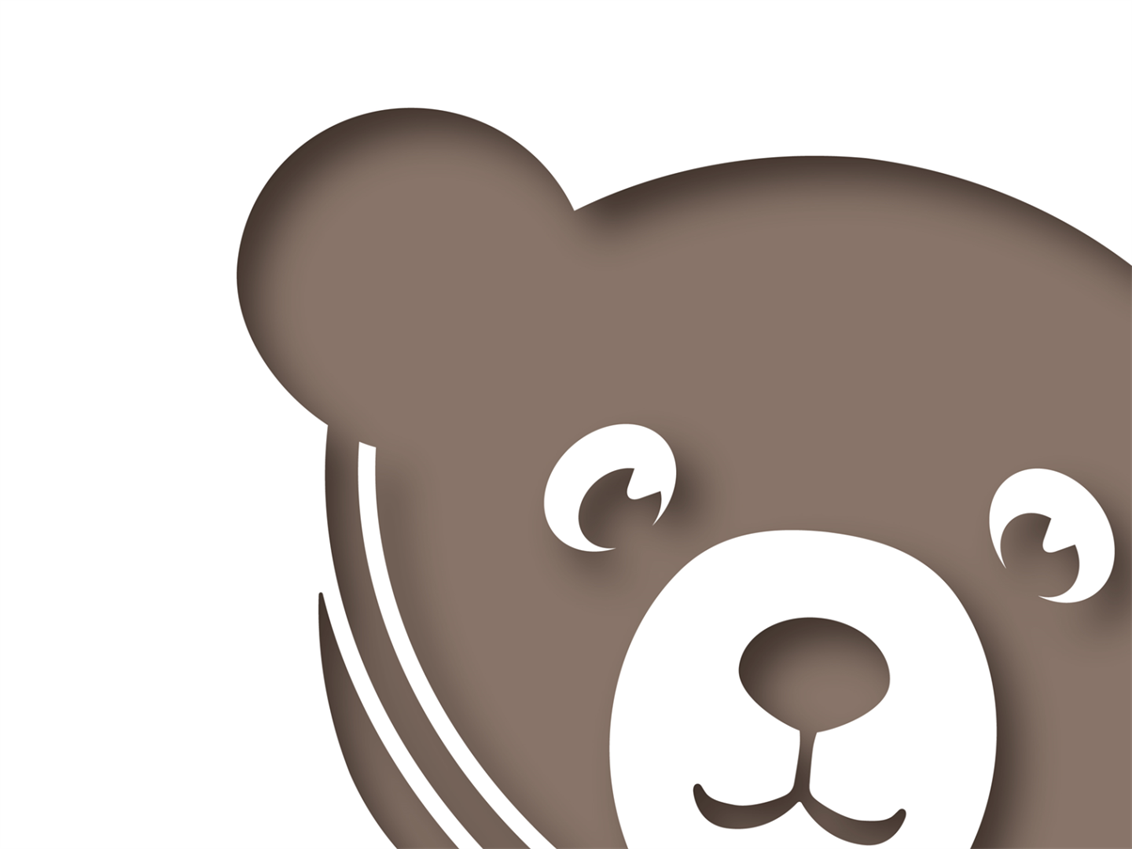

Supplement: Supplementary file 1 — CA-TIC Premodule folderCA-TIC Module 1 folderCA-TIC Module 2 folderCA-TIC Module 3 folderImage Citations.docxCA-TIC Evaluation.docx [file mep_2374-8265.10990-s001.zip › A. CA-TIC Premodule/mobile/5wMscbCqwuC_DX1440_DY1440_CX1132_CY849.png]

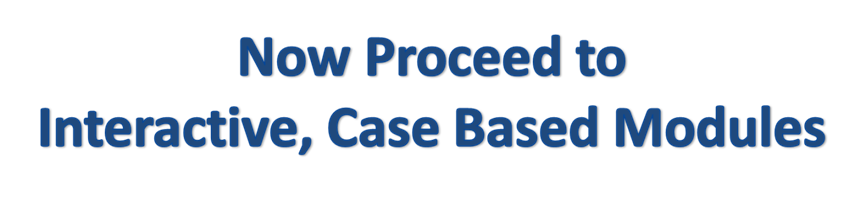

Supplement: Supplementary file 1 — CA-TIC Premodule folderCA-TIC Module 1 folderCA-TIC Module 2 folderCA-TIC Module 3 folderImage Citations.docxCA-TIC Evaluation.docx [file mep_2374-8265.10990-s001.zip › A. CA-TIC Premodule/mobile/65Qy8iYCM16_DX1388_DY1388_CX867_CY201.png]

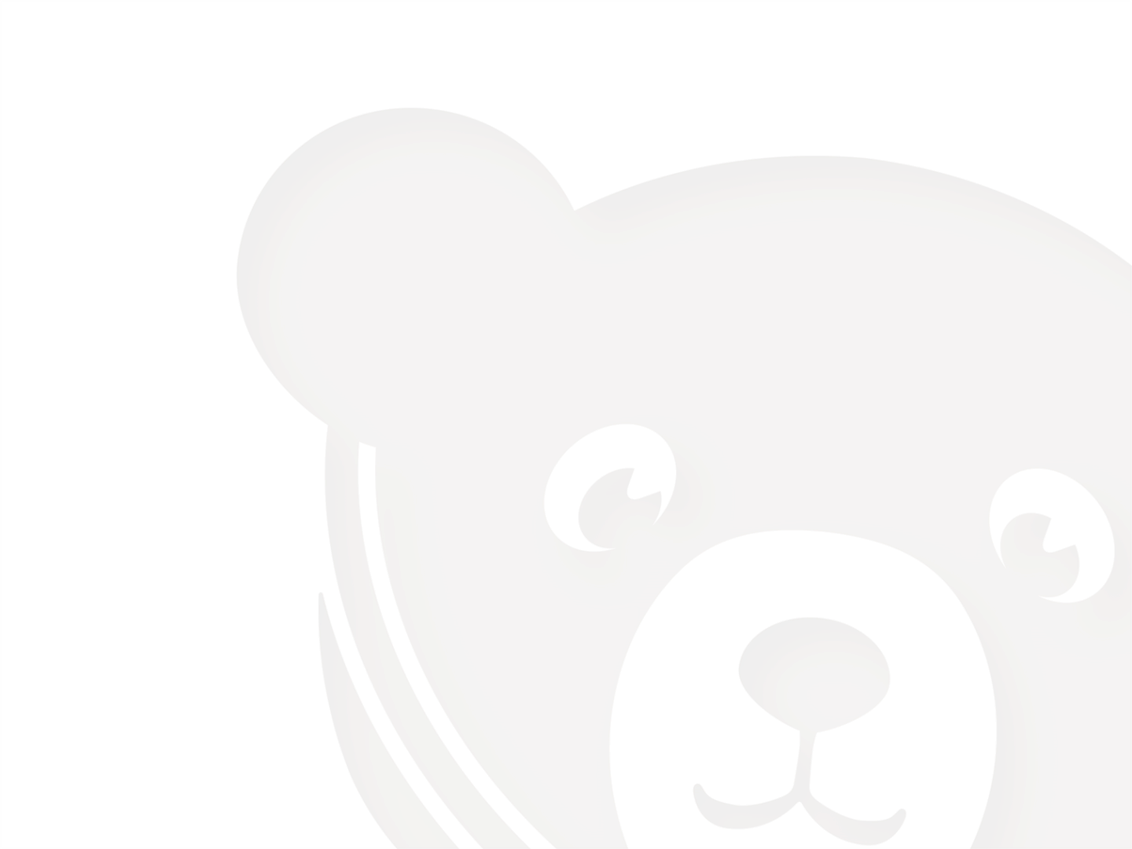

Supplement: Supplementary file 1 — CA-TIC Premodule folderCA-TIC Module 1 folderCA-TIC Module 2 folderCA-TIC Module 3 folderImage Citations.docxCA-TIC Evaluation.docx [file mep_2374-8265.10990-s001.zip › A. CA-TIC Premodule/mobile/6ENtf7OFty5_DX1440_DY1440_CX1132_CY849.png]

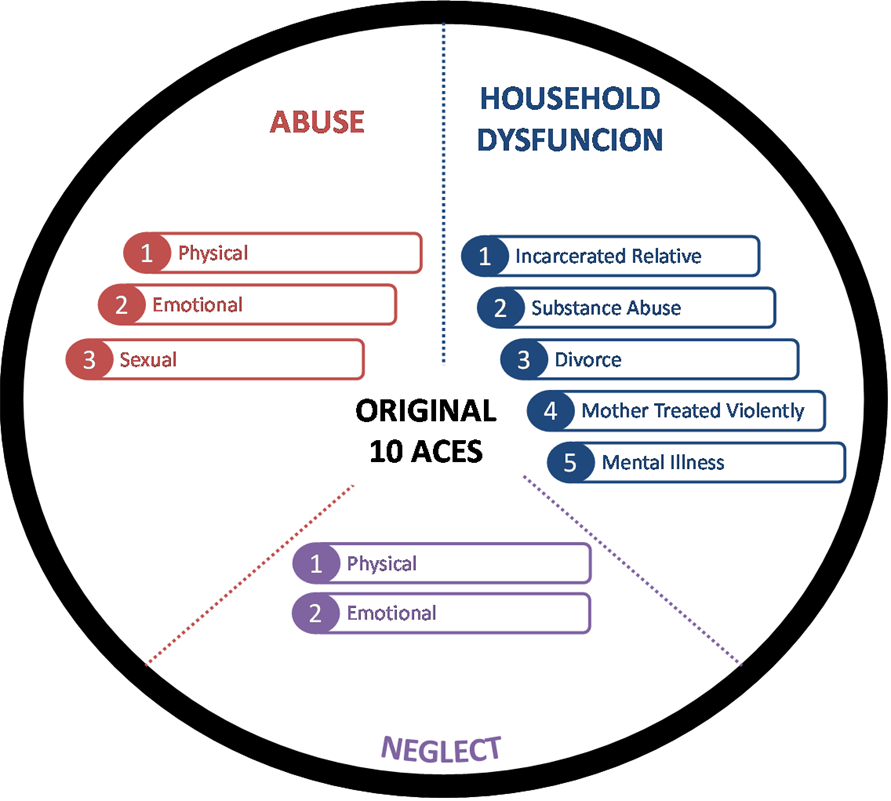

Supplement: Supplementary file 1 — CA-TIC Premodule folderCA-TIC Module 1 folderCA-TIC Module 2 folderCA-TIC Module 3 folderImage Citations.docxCA-TIC Evaluation.docx [file mep_2374-8265.10990-s001.zip › A. CA-TIC Premodule/mobile/6GS49z6Av0t_DX1136_DY1136_CX891_CY801.png]

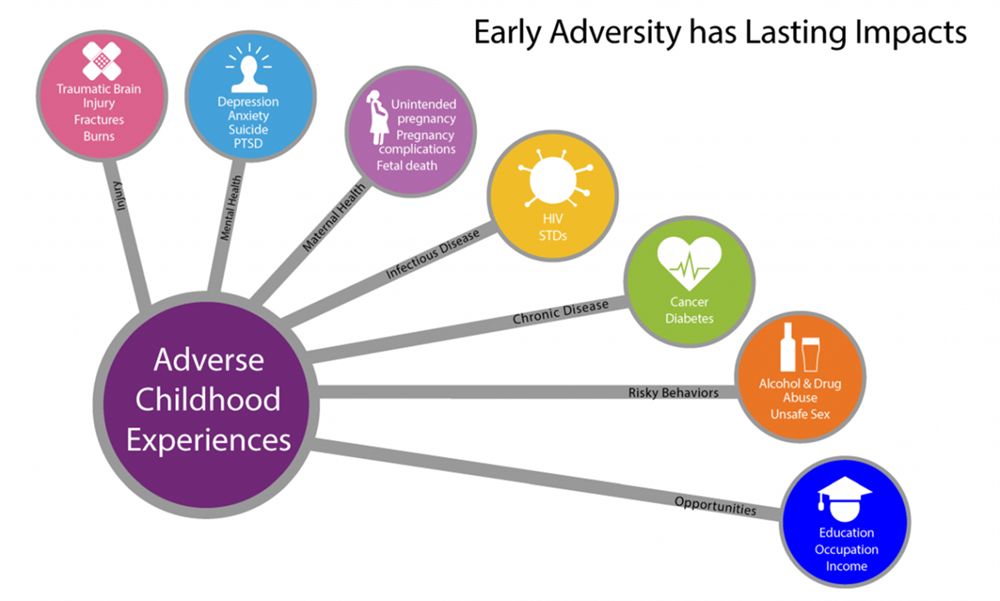

Supplement: Supplementary file 1 — CA-TIC Premodule folderCA-TIC Module 1 folderCA-TIC Module 2 folderCA-TIC Module 3 folderImage Citations.docxCA-TIC Evaluation.docx [file mep_2374-8265.10990-s001.zip › A. CA-TIC Premodule/mobile/6I3J2z1NVfm_DX1274_DY1274_CX1001_CY601.png]

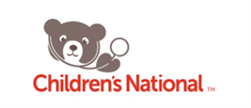

Supplement: Supplementary file 1 — CA-TIC Premodule folderCA-TIC Module 1 folderCA-TIC Module 2 folderCA-TIC Module 3 folderImage Citations.docxCA-TIC Evaluation.docx [file mep_2374-8265.10990-s001.zip › A. CA-TIC Premodule/mobile/6qq59COusIr_DX322_DY322_CX252_CY108.png]

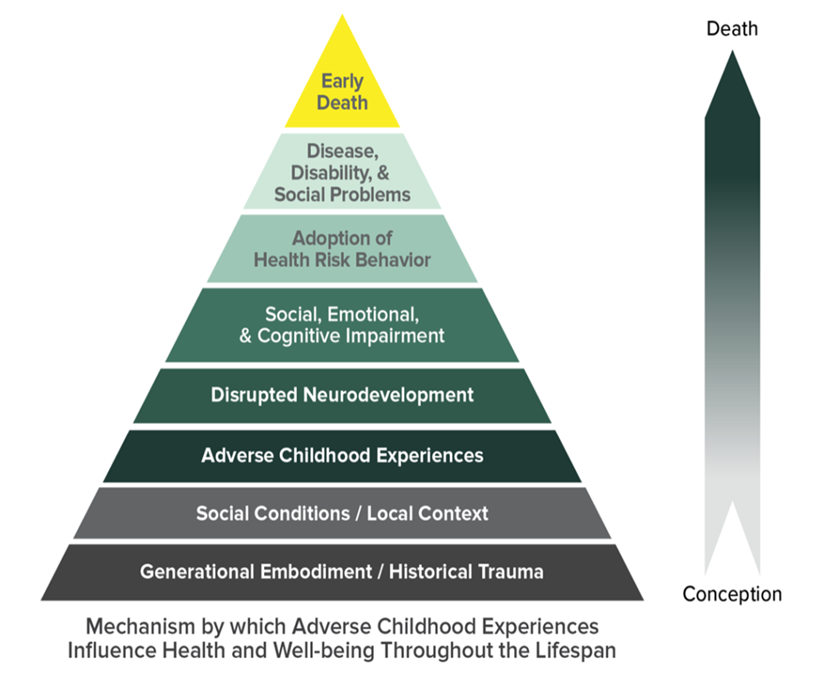

Supplement: Supplementary file 1 — CA-TIC Premodule folderCA-TIC Module 1 folderCA-TIC Module 2 folderCA-TIC Module 3 folderImage Citations.docxCA-TIC Evaluation.docx [file mep_2374-8265.10990-s001.zip › A. CA-TIC Premodule/mobile/6XCEbB8rqjJ_DX1048_DY1048_CX824_CY690.png]

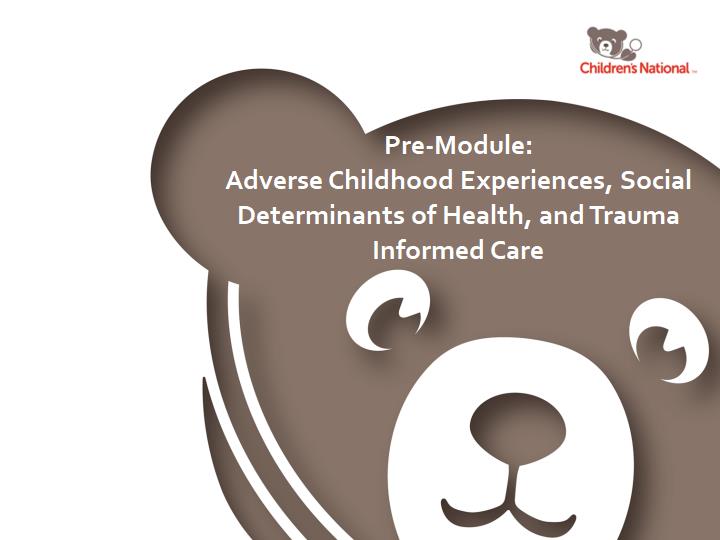

Supplement: Supplementary file 1 — CA-TIC Premodule folderCA-TIC Module 1 folderCA-TIC Module 2 folderCA-TIC Module 3 folderImage Citations.docxCA-TIC Evaluation.docx [file mep_2374-8265.10990-s001.zip › A. CA-TIC Premodule/story_content/thumbnail.jpg]

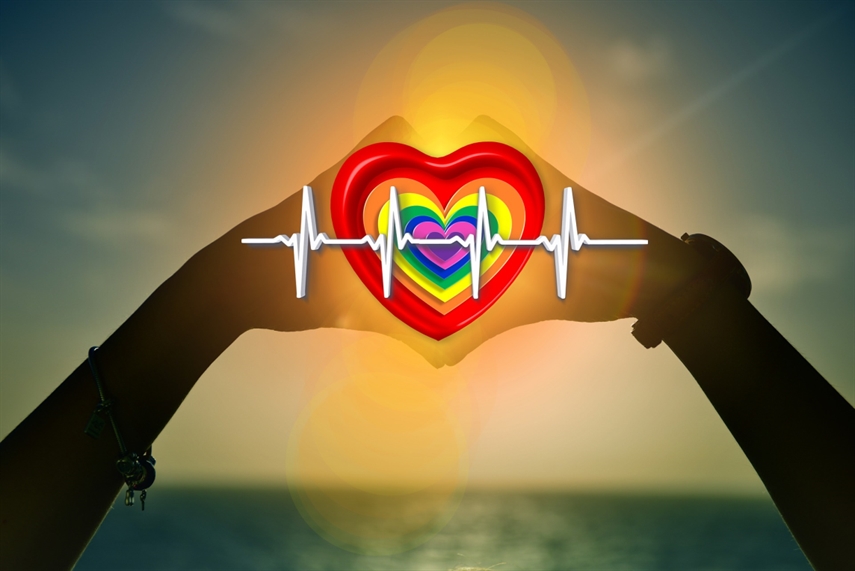

Supplement: Supplementary file 1 — CA-TIC Premodule folderCA-TIC Module 1 folderCA-TIC Module 2 folderCA-TIC Module 3 folderImage Citations.docxCA-TIC Evaluation.docx [file mep_2374-8265.10990-s001.zip › B. CA-TIC Module 1/mobile/5gjH5muHmn6_DX1144_DY1144_CX856_CY571.jpg]

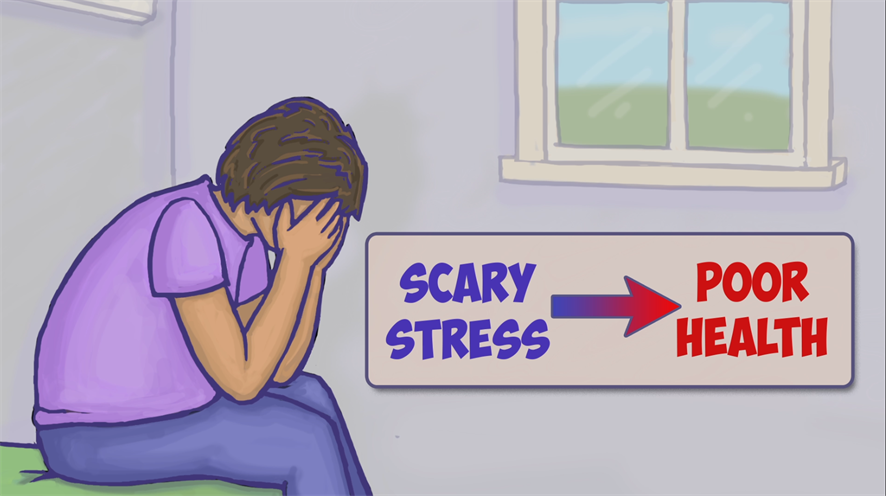

Supplement: Supplementary file 1 — CA-TIC Premodule folderCA-TIC Module 1 folderCA-TIC Module 2 folderCA-TIC Module 3 folderImage Citations.docxCA-TIC Evaluation.docx [file mep_2374-8265.10990-s001.zip › B. CA-TIC Module 1/mobile/5nbQy6gxisr_P_8_9_1776_995_DX1186_DY1186_CX886_CY497.png]

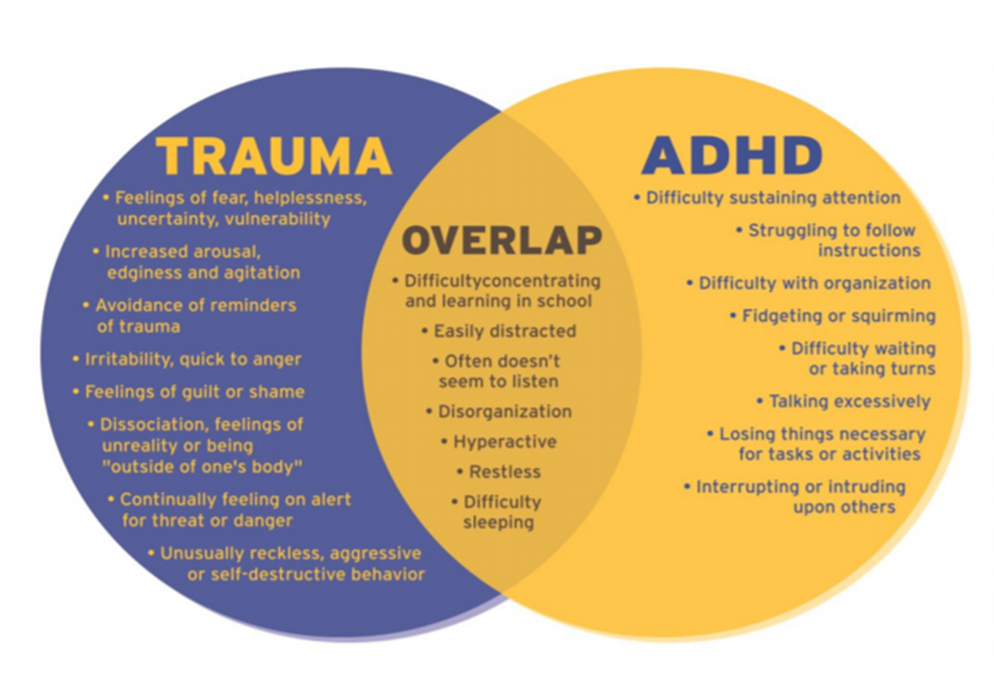

Supplement: Supplementary file 1 — CA-TIC Premodule folderCA-TIC Module 1 folderCA-TIC Module 2 folderCA-TIC Module 3 folderImage Citations.docxCA-TIC Evaluation.docx [file mep_2374-8265.10990-s001.zip › B. CA-TIC Module 1/mobile/5peQaeuXK6B_P_75_333_4306_2979_DX1356_DY1356_CX994_CY688.png]

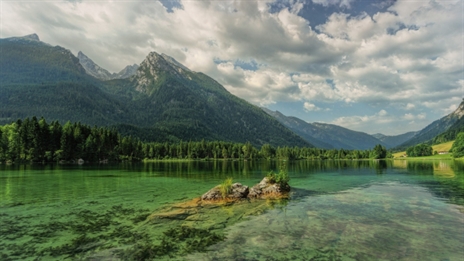

Supplement: Supplementary file 1 — CA-TIC Premodule folderCA-TIC Module 1 folderCA-TIC Module 2 folderCA-TIC Module 3 folderImage Citations.docxCA-TIC Evaluation.docx [file mep_2374-8265.10990-s001.zip › B. CA-TIC Module 1/mobile/5XeSohPHO9G_DX620_DY620_CX464_CY261.jpg]

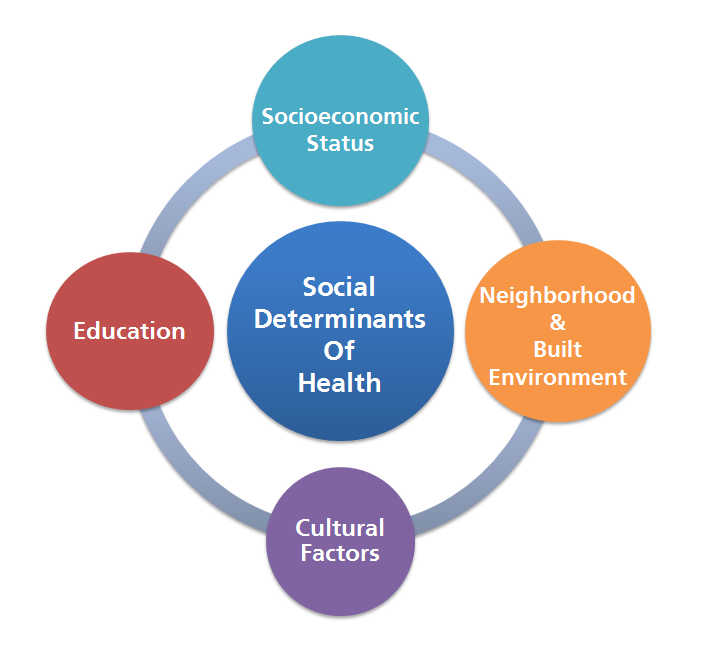

Supplement: Supplementary file 1 — CA-TIC Premodule folderCA-TIC Module 1 folderCA-TIC Module 2 folderCA-TIC Module 3 folderImage Citations.docxCA-TIC Evaluation.docx [file mep_2374-8265.10990-s001.zip › B. CA-TIC Module 1/mobile/5xQF17EOjtq_DX1134_DY1134_CX704_CY654.png]

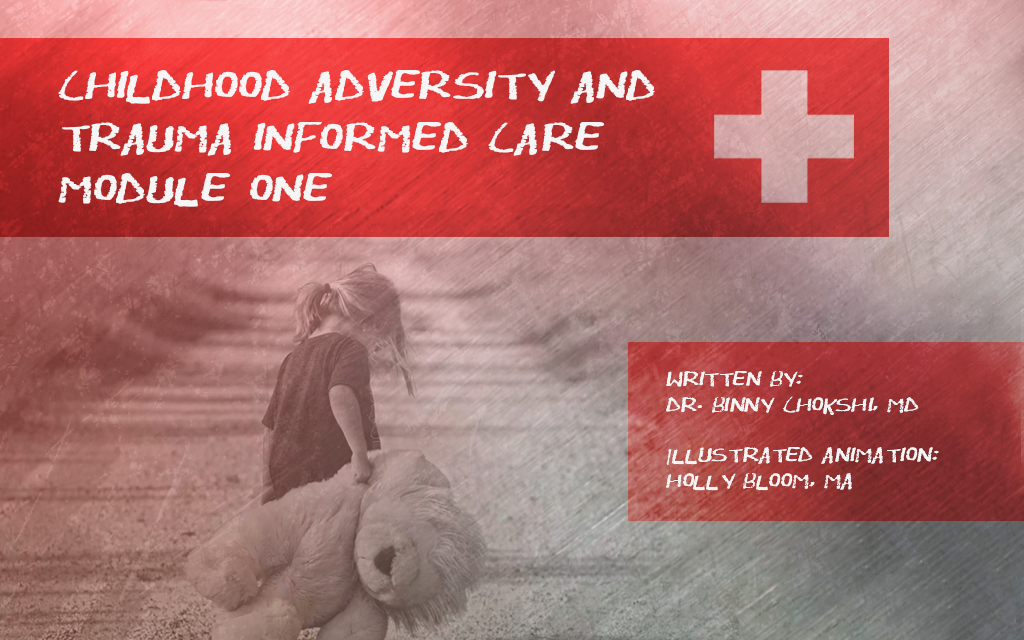

Supplement: Supplementary file 1 — CA-TIC Premodule folderCA-TIC Module 1 folderCA-TIC Module 2 folderCA-TIC Module 3 folderImage Citations.docxCA-TIC Evaluation.docx [file mep_2374-8265.10990-s001.zip › B. CA-TIC Module 1/mobile/5YTAIFUfvVv_DX2048_DY2048_CX1024_CY640.png]

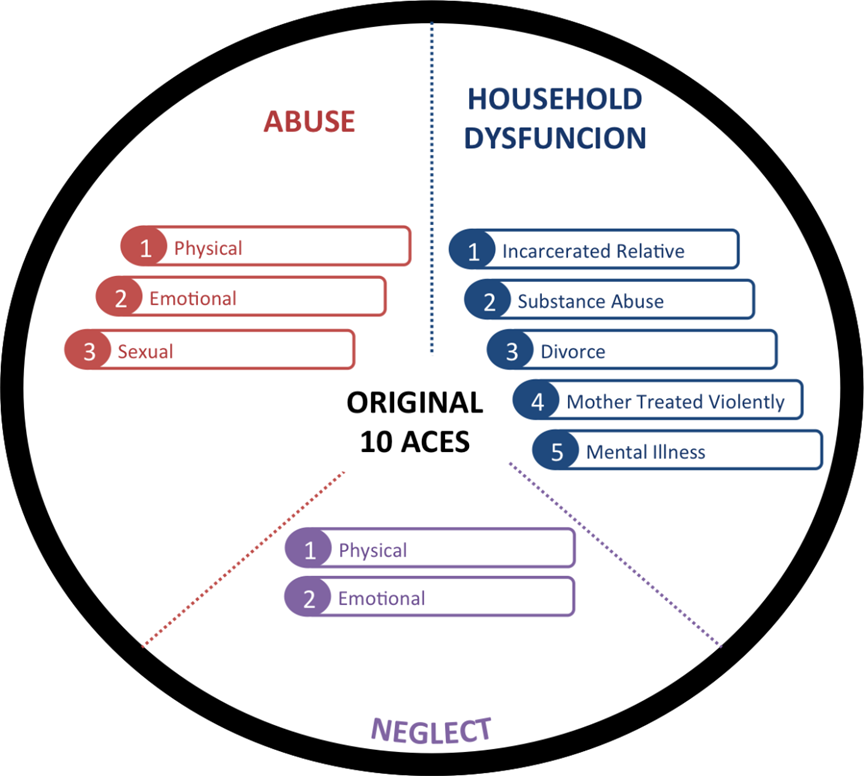

Supplement: Supplementary file 1 — CA-TIC Premodule folderCA-TIC Module 1 folderCA-TIC Module 2 folderCA-TIC Module 3 folderImage Citations.docxCA-TIC Evaluation.docx [file mep_2374-8265.10990-s001.zip › B. CA-TIC Module 1/mobile/61G2vQmEyOn_DX1154_DY1154_CX865_CY776.png]

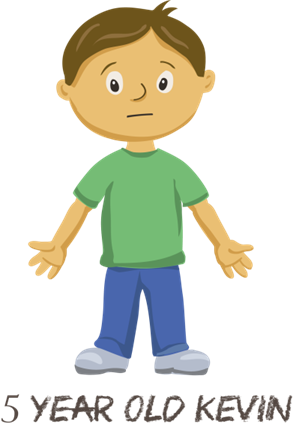

Supplement: Supplementary file 1 — CA-TIC Premodule folderCA-TIC Module 1 folderCA-TIC Module 2 folderCA-TIC Module 3 folderImage Citations.docxCA-TIC Evaluation.docx [file mep_2374-8265.10990-s001.zip › B. CA-TIC Module 1/mobile/69DQgaq9Lk2_DX574_DY574_CX293_CY430.png]

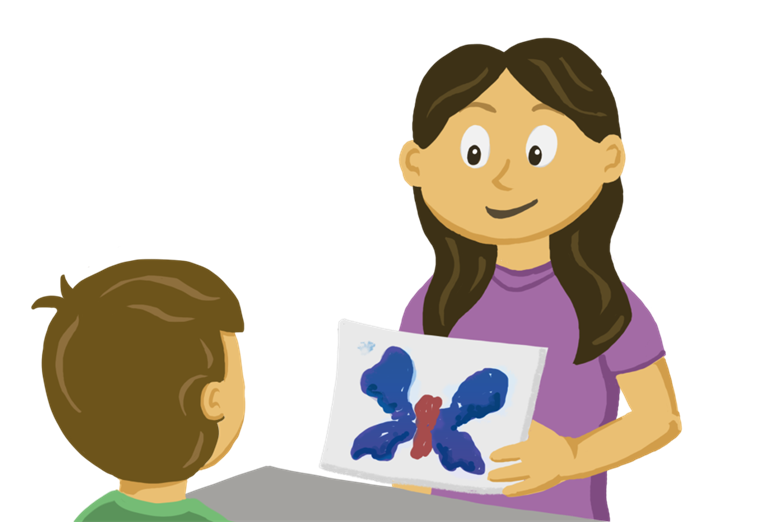

Supplement: Supplementary file 1 — CA-TIC Premodule folderCA-TIC Module 1 folderCA-TIC Module 2 folderCA-TIC Module 3 folderImage Citations.docxCA-TIC Evaluation.docx [file mep_2374-8265.10990-s001.zip › B. CA-TIC Module 1/mobile/6JQ2yIiUn0G_DX1044_DY1044_CX783_CY522.png]

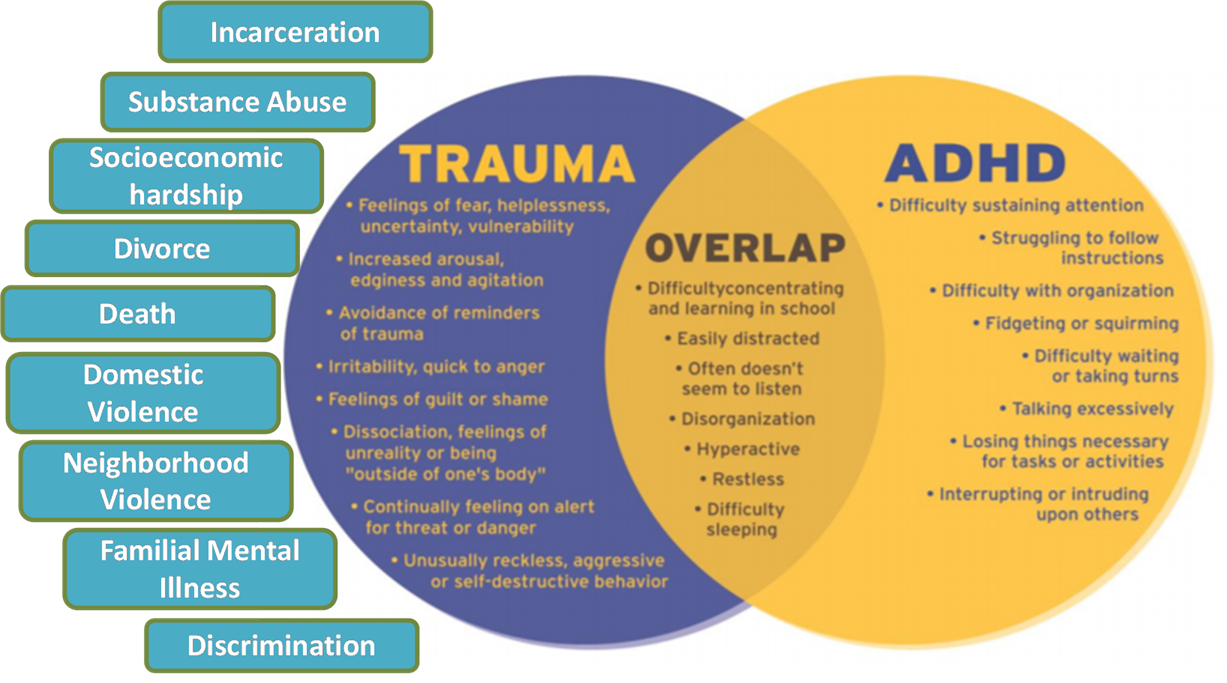

Supplement: Supplementary file 1 — CA-TIC Premodule folderCA-TIC Module 1 folderCA-TIC Module 2 folderCA-TIC Module 3 folderImage Citations.docxCA-TIC Evaluation.docx [file mep_2374-8265.10990-s001.zip › B. CA-TIC Module 1/mobile/6kCGwPTm12N_DX1636_DY1636_CX1227_CY684.png]

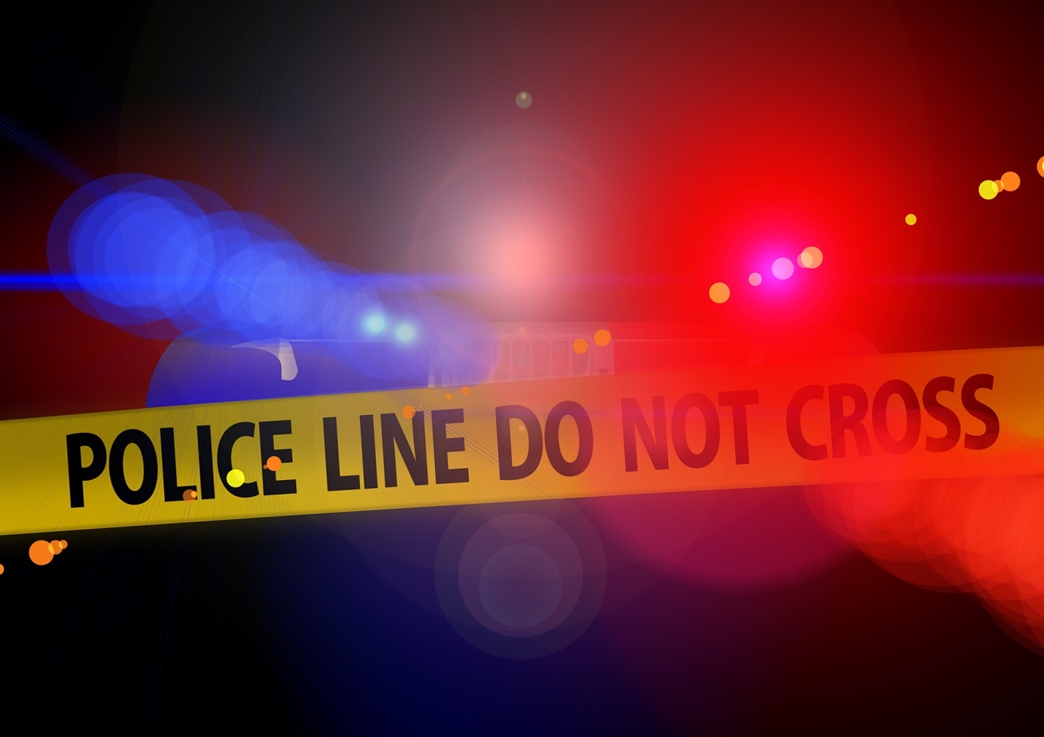

Supplement: Supplementary file 1 — CA-TIC Premodule folderCA-TIC Module 1 folderCA-TIC Module 2 folderCA-TIC Module 3 folderImage Citations.docxCA-TIC Evaluation.docx [file mep_2374-8265.10990-s001.zip › B. CA-TIC Module 1/mobile/6Kom4YCcSDj_DX1392_DY1392_CX1044_CY738.jpg]

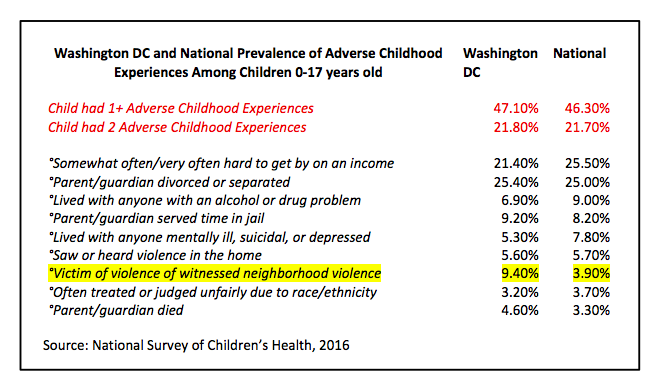

Supplement: Supplementary file 1 — CA-TIC Premodule folderCA-TIC Module 1 folderCA-TIC Module 2 folderCA-TIC Module 3 folderImage Citations.docxCA-TIC Evaluation.docx [file mep_2374-8265.10990-s001.zip › B. CA-TIC Module 1/mobile/6n2DXN6e2yF_DX1312_DY1312_CX656_CY389.png]

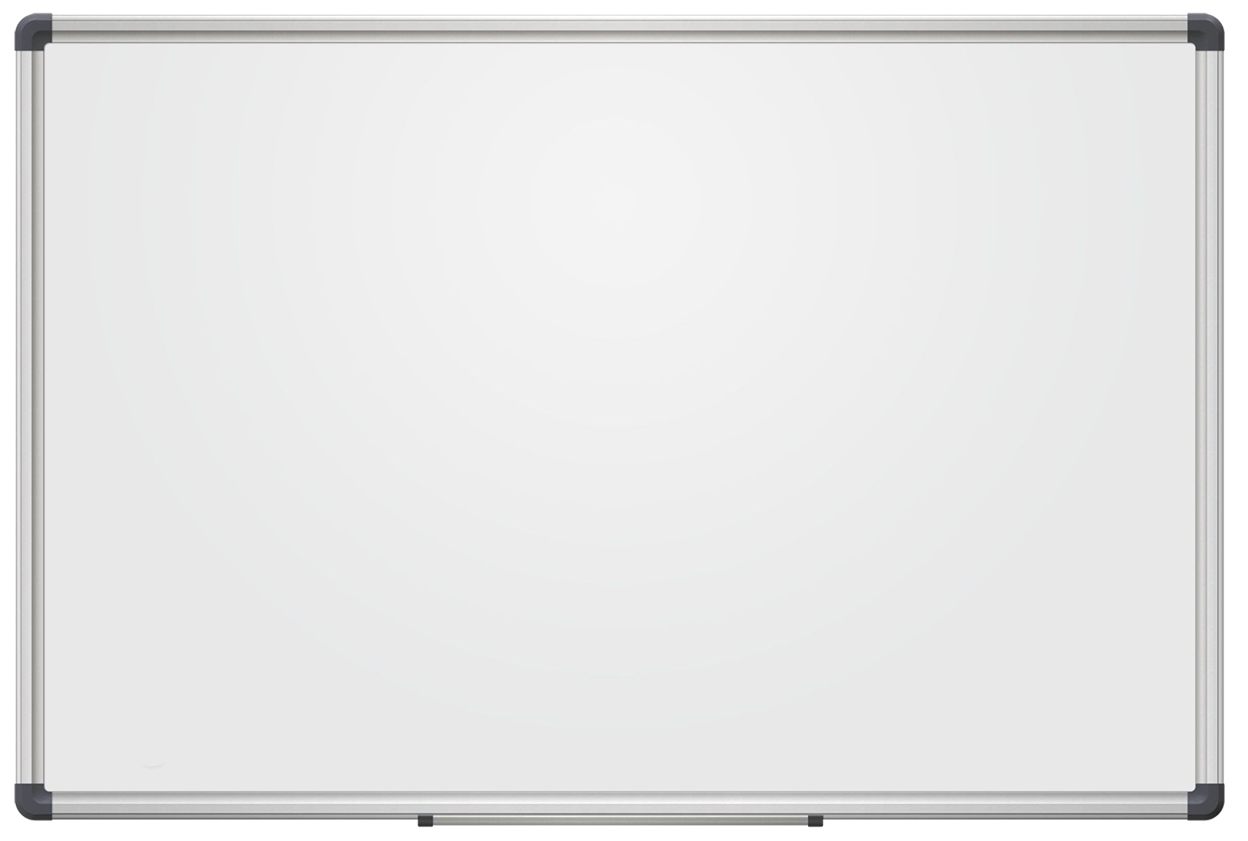

Supplement: Supplementary file 1 — CA-TIC Premodule folderCA-TIC Module 1 folderCA-TIC Module 2 folderCA-TIC Module 3 folderImage Citations.docxCA-TIC Evaluation.docx [file mep_2374-8265.10990-s001.zip › B. CA-TIC Module 1/mobile/6nAYD602p6V_DX1762_DY1762_CX1248_CY841.png]

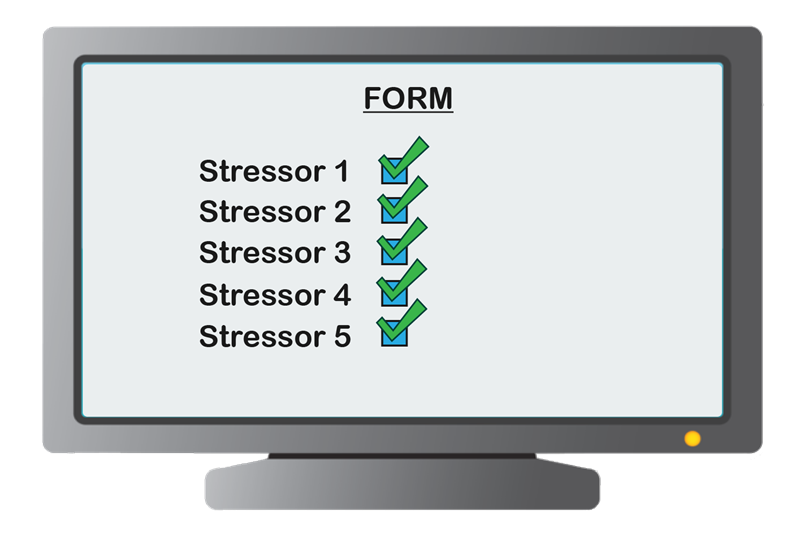

Supplement: Supplementary file 1 — CA-TIC Premodule folderCA-TIC Module 1 folderCA-TIC Module 2 folderCA-TIC Module 3 folderImage Citations.docxCA-TIC Evaluation.docx [file mep_2374-8265.10990-s001.zip › B. CA-TIC Module 1/mobile/6nO57pikDNV_P_119_335_1545_1045_DX1238_DY1238_CX791_CY535.png]

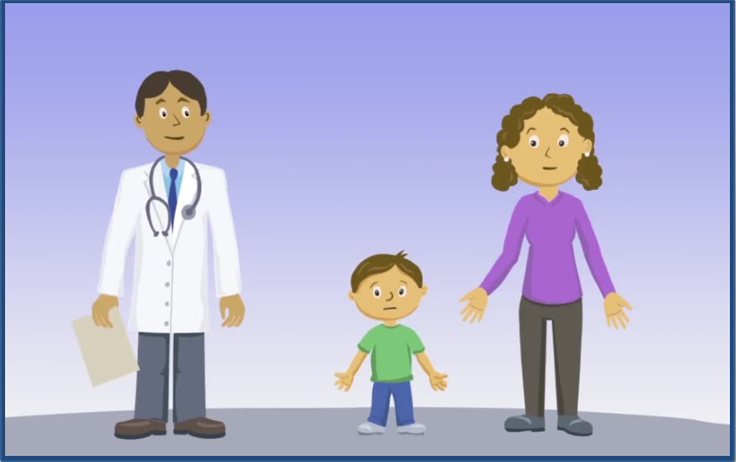

Supplement: Supplementary file 1 — CA-TIC Premodule folderCA-TIC Module 1 folderCA-TIC Module 2 folderCA-TIC Module 3 folderImage Citations.docxCA-TIC Evaluation.docx [file mep_2374-8265.10990-s001.zip › B. CA-TIC Module 1/mobile/6pDx8oVLMON_DX1472_DY1472_CX736_CY462.png]

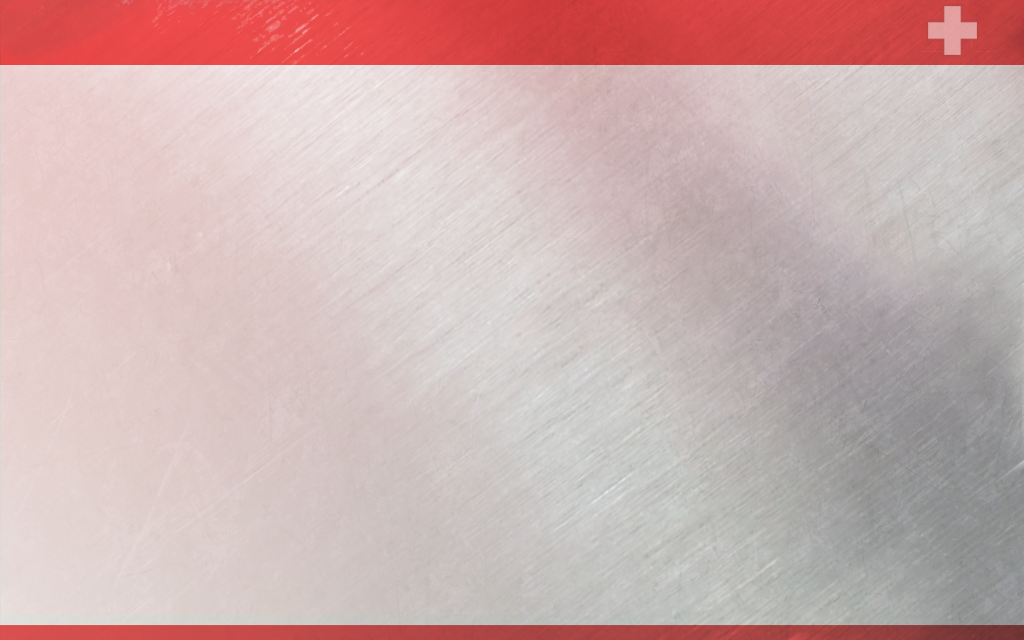

Supplement: Supplementary file 1 — CA-TIC Premodule folderCA-TIC Module 1 folderCA-TIC Module 2 folderCA-TIC Module 3 folderImage Citations.docxCA-TIC Evaluation.docx [file mep_2374-8265.10990-s001.zip › B. CA-TIC Module 1/mobile/6pj5DG5xGK8_DX2048_DY2048_CX1024_CY640.png]

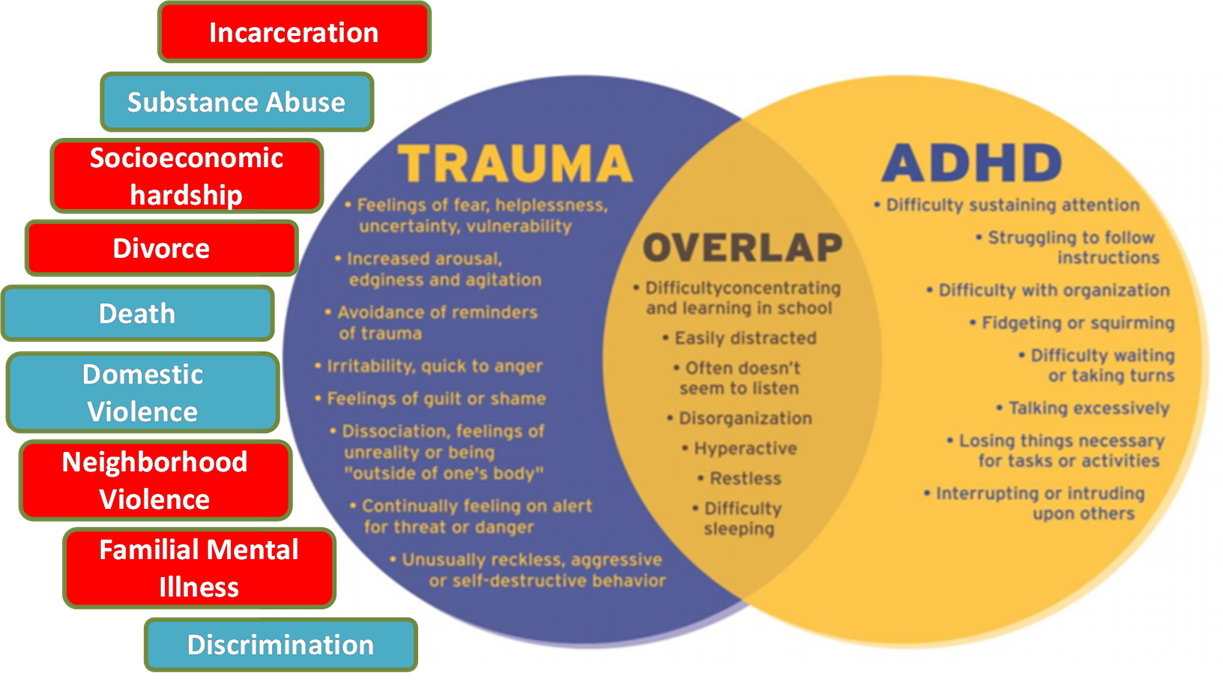

Supplement: Supplementary file 1 — CA-TIC Premodule folderCA-TIC Module 1 folderCA-TIC Module 2 folderCA-TIC Module 3 folderImage Citations.docxCA-TIC Evaluation.docx [file mep_2374-8265.10990-s001.zip › B. CA-TIC Module 1/mobile/6T73T4GloJw_DX1632_DY1632_CX1224_CY682.png]

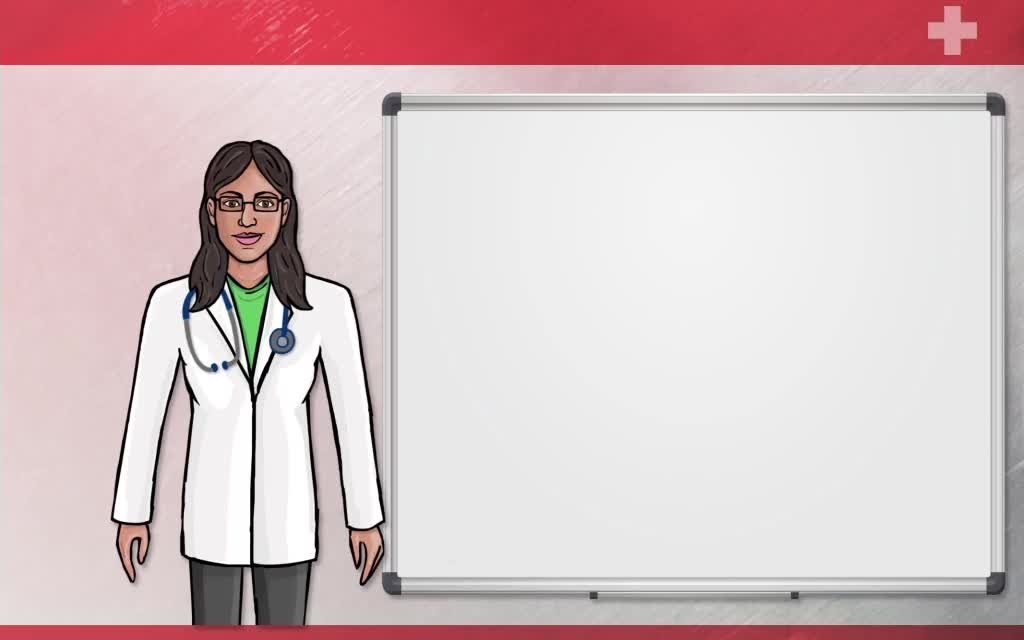

Supplement: Supplementary file 1 — CA-TIC Premodule folderCA-TIC Module 1 folderCA-TIC Module 2 folderCA-TIC Module 3 folderImage Citations.docxCA-TIC Evaluation.docx [file mep_2374-8265.10990-s001.zip › B. CA-TIC Module 1/mobile/poster_5sPSiVgotJW_video_6EDNnqtTVsv_18_48_1024x640.jpg]

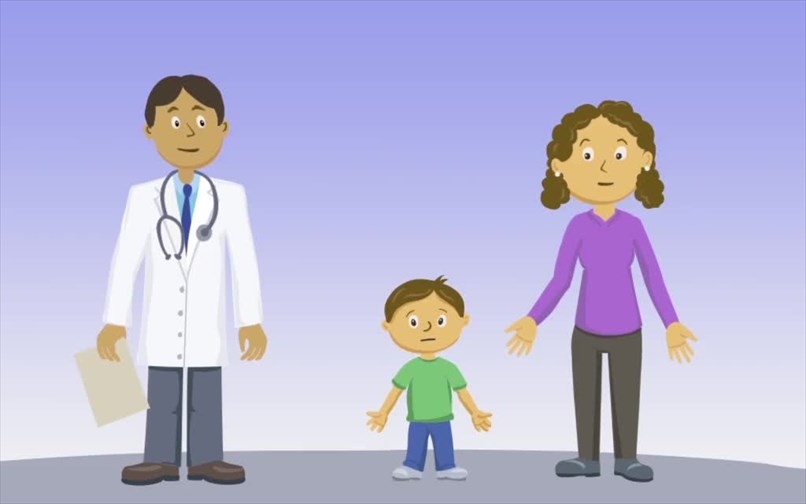

Supplement: Supplementary file 1 — CA-TIC Premodule folderCA-TIC Module 1 folderCA-TIC Module 2 folderCA-TIC Module 3 folderImage Citations.docxCA-TIC Evaluation.docx [file mep_2374-8265.10990-s001.zip › B. CA-TIC Module 1/mobile/poster_5WYF9PgFs66_video_5hH5Xqj7Kv0_18_48_806x504.jpg]

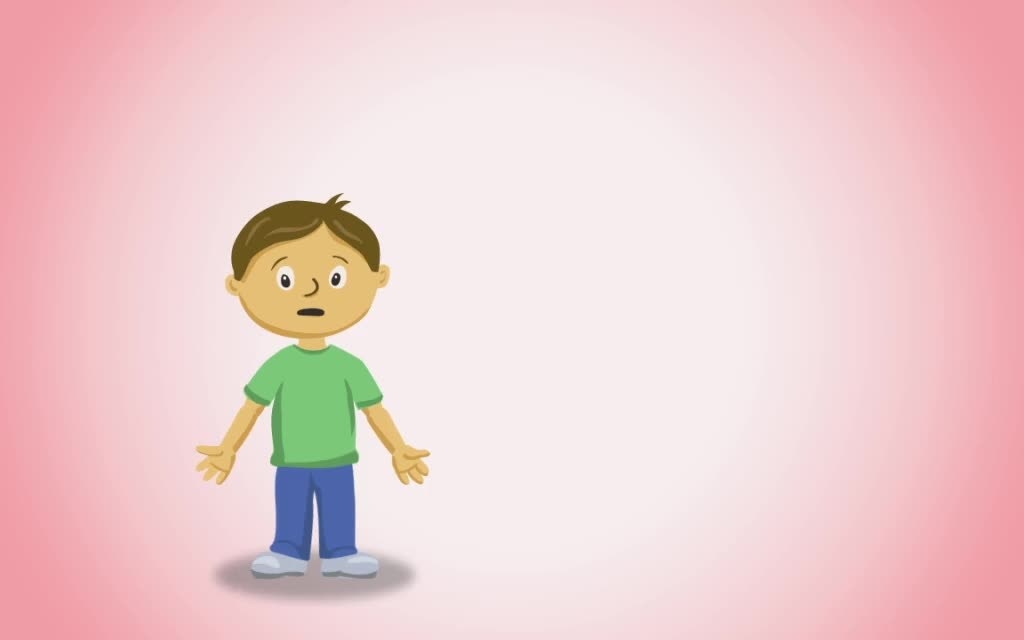

Supplement: Supplementary file 1 — CA-TIC Premodule folderCA-TIC Module 1 folderCA-TIC Module 2 folderCA-TIC Module 3 folderImage Citations.docxCA-TIC Evaluation.docx [file mep_2374-8265.10990-s001.zip › B. CA-TIC Module 1/mobile/poster_6EVM5lrX9iF_video_6pkse7762nI_18_48_858x536.jpg]

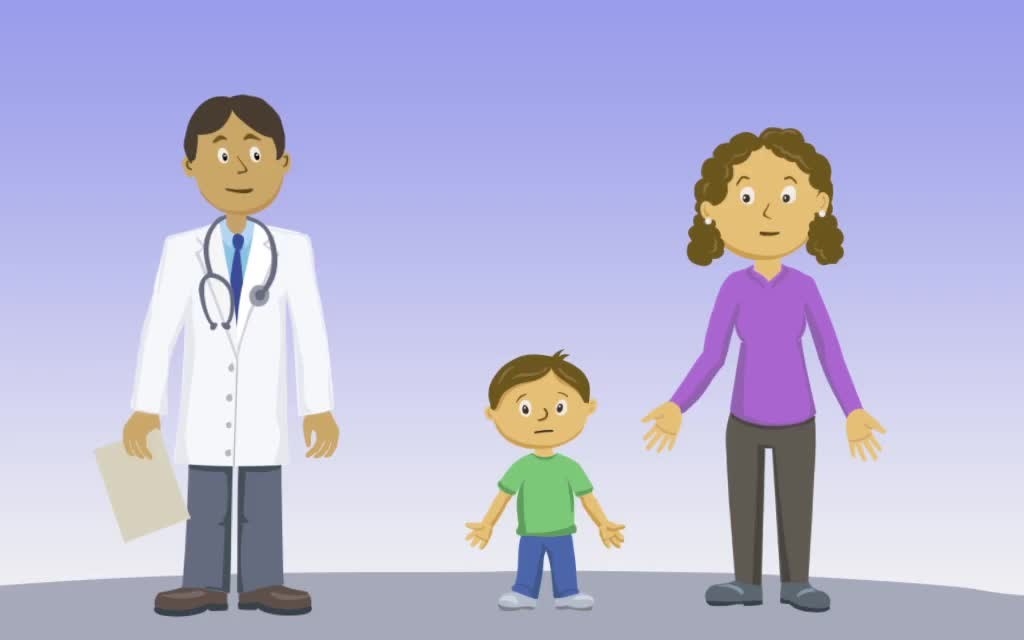

Supplement: Supplementary file 1 — CA-TIC Premodule folderCA-TIC Module 1 folderCA-TIC Module 2 folderCA-TIC Module 3 folderImage Citations.docxCA-TIC Evaluation.docx [file mep_2374-8265.10990-s001.zip › B. CA-TIC Module 1/mobile/poster_6LeVLfVPiyM_video_6ijBptOvhuw_18_48_806x504.jpg]

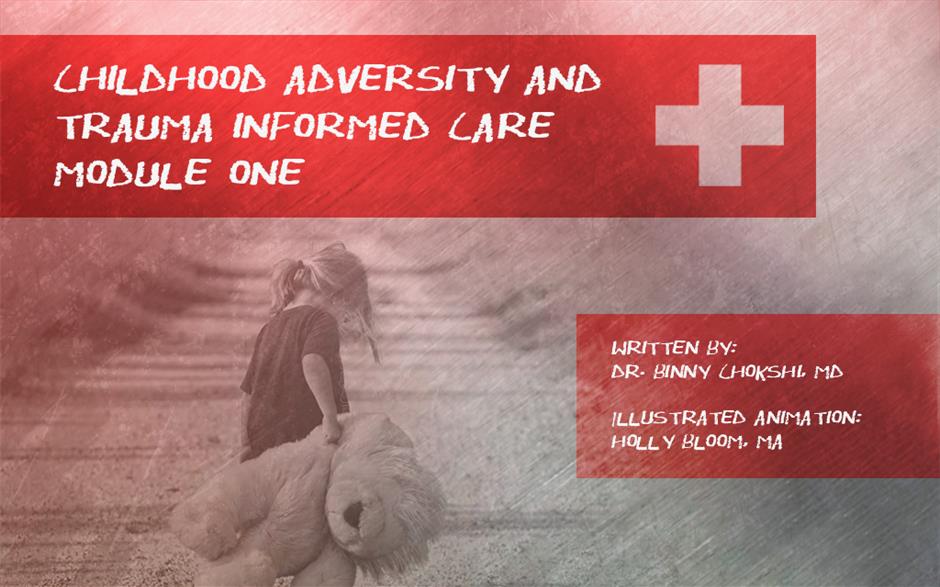

Supplement: Supplementary file 1 — CA-TIC Premodule folderCA-TIC Module 1 folderCA-TIC Module 2 folderCA-TIC Module 3 folderImage Citations.docxCA-TIC Evaluation.docx [file mep_2374-8265.10990-s001.zip › B. CA-TIC Module 1/story_content/thumbnail.jpg]

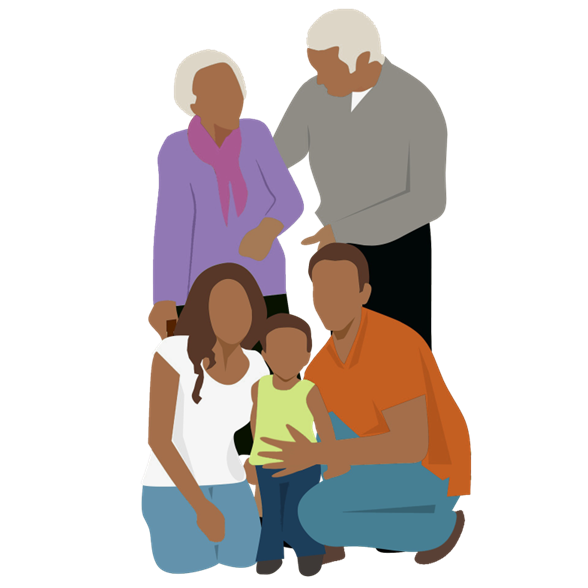

Supplement: Supplementary file 1 — CA-TIC Premodule folderCA-TIC Module 1 folderCA-TIC Module 2 folderCA-TIC Module 3 folderImage Citations.docxCA-TIC Evaluation.docx [file mep_2374-8265.10990-s001.zip › C. CA-TIC Module 2/mobile/5g9ryIXfk2X_DX784_DY784_CX588_CY588.png]

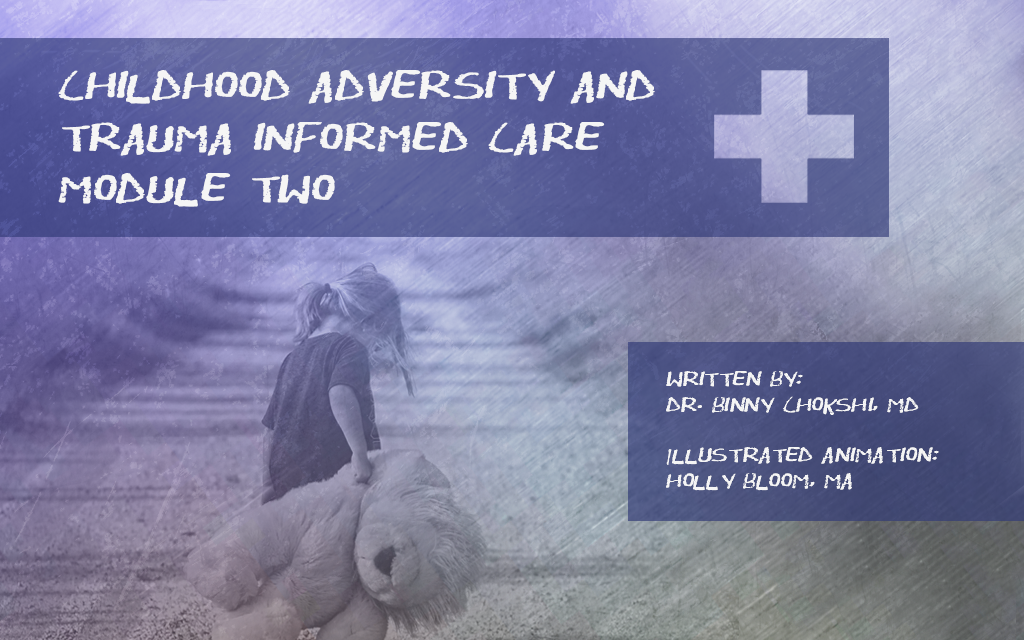

Supplement: Supplementary file 1 — CA-TIC Premodule folderCA-TIC Module 1 folderCA-TIC Module 2 folderCA-TIC Module 3 folderImage Citations.docxCA-TIC Evaluation.docx [file mep_2374-8265.10990-s001.zip › C. CA-TIC Module 2/mobile/5nIFKaTVf81_DX2048_DY2048_CX1024_CY640.png]

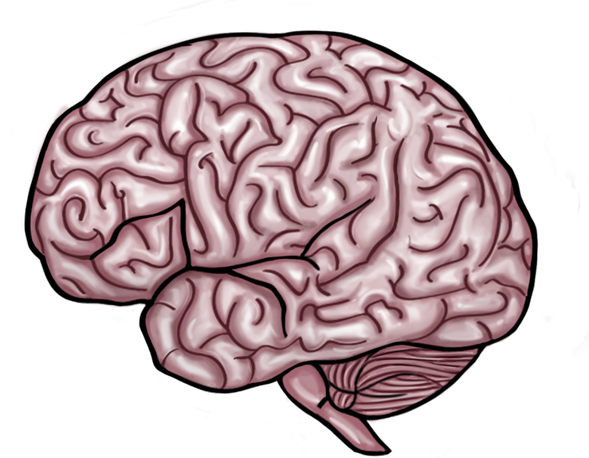

Supplement: Supplementary file 1 — CA-TIC Premodule folderCA-TIC Module 1 folderCA-TIC Module 2 folderCA-TIC Module 3 folderImage Citations.docxCA-TIC Evaluation.docx [file mep_2374-8265.10990-s001.zip › C. CA-TIC Module 2/mobile/5nLIYqVQyVG_DX786_DY786_CX589_CY458.png]

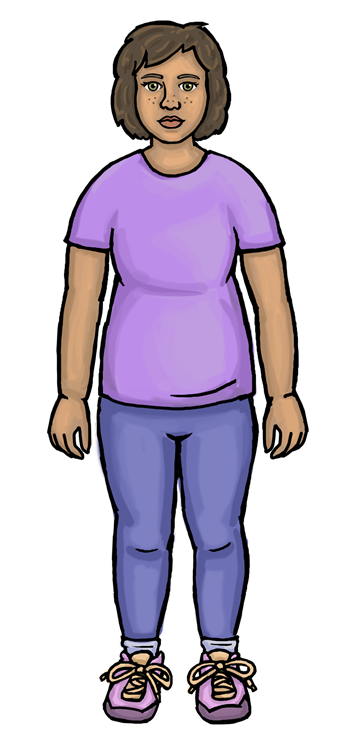

Supplement: Supplementary file 1 — CA-TIC Premodule folderCA-TIC Module 1 folderCA-TIC Module 2 folderCA-TIC Module 3 folderImage Citations.docxCA-TIC Evaluation.docx [file mep_2374-8265.10990-s001.zip › C. CA-TIC Module 2/mobile/5oq4DX9W8eE_P_0_0_958_2020_DX996_DY996_CX349_CY736.png]

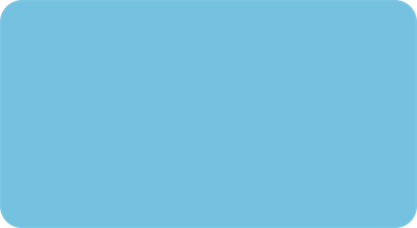

Supplement: Supplementary file 1 — CA-TIC Premodule folderCA-TIC Module 1 folderCA-TIC Module 2 folderCA-TIC Module 3 folderImage Citations.docxCA-TIC Evaluation.docx [file mep_2374-8265.10990-s001.zip › C. CA-TIC Module 2/mobile/5owqjCkf3nh_DX604_DY604_CX418_CY228.png]

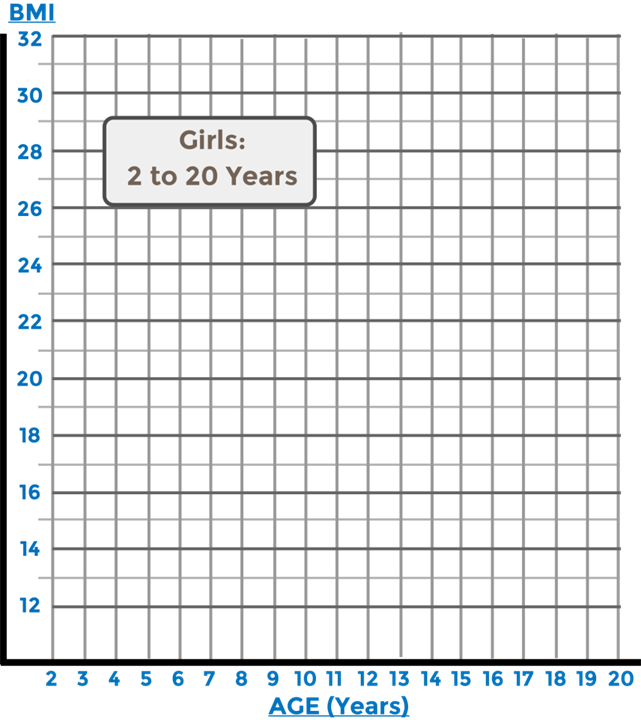

Supplement: Supplementary file 1 — CA-TIC Premodule folderCA-TIC Module 1 folderCA-TIC Module 2 folderCA-TIC Module 3 folderImage Citations.docxCA-TIC Evaluation.docx [file mep_2374-8265.10990-s001.zip › C. CA-TIC Module 2/mobile/5rB174x3zIF_DX960_DY960_CX642_CY720.png]

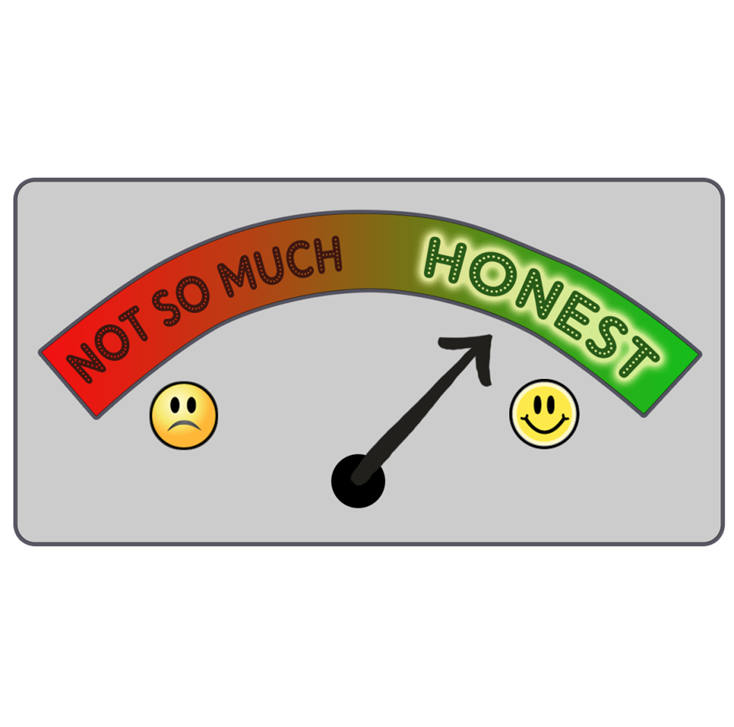

Supplement: Supplementary file 1 — CA-TIC Premodule folderCA-TIC Module 1 folderCA-TIC Module 2 folderCA-TIC Module 3 folderImage Citations.docxCA-TIC Evaluation.docx [file mep_2374-8265.10990-s001.zip › C. CA-TIC Module 2/mobile/5rHMJlg4sBF_DX980_DY980_CX735_CY727.png]

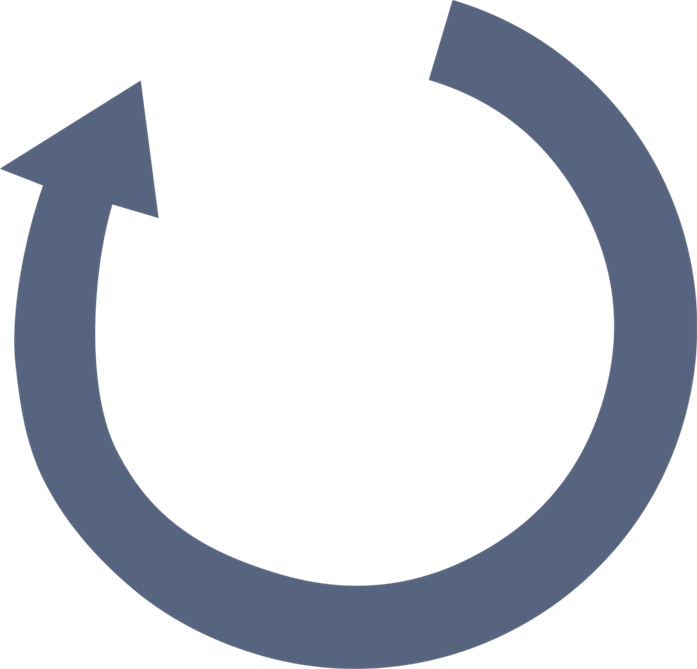

Supplement: Supplementary file 1 — CA-TIC Premodule folderCA-TIC Module 1 folderCA-TIC Module 2 folderCA-TIC Module 3 folderImage Citations.docxCA-TIC Evaluation.docx [file mep_2374-8265.10990-s001.zip › C. CA-TIC Module 2/mobile/5wgkqCZAdI7_DX930_DY930_CX697_CY669.png]

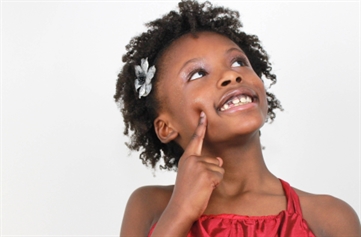

Supplement: Supplementary file 1 — CA-TIC Premodule folderCA-TIC Module 1 folderCA-TIC Module 2 folderCA-TIC Module 3 folderImage Citations.docxCA-TIC Evaluation.docx [file mep_2374-8265.10990-s001.zip › C. CA-TIC Module 2/mobile/5wlHrg7QU5g_DX482_DY482_CX361_CY238.jpg]

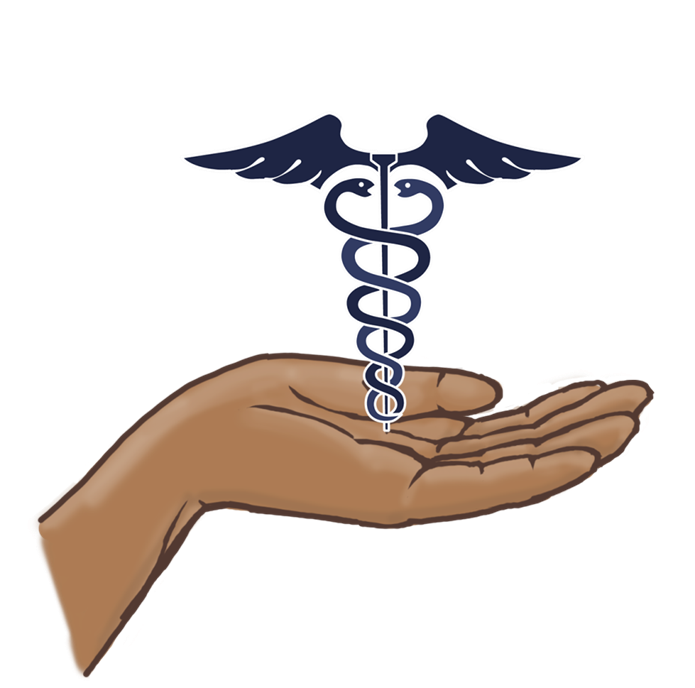

Supplement: Supplementary file 1 — CA-TIC Premodule folderCA-TIC Module 1 folderCA-TIC Module 2 folderCA-TIC Module 3 folderImage Citations.docxCA-TIC Evaluation.docx [file mep_2374-8265.10990-s001.zip › C. CA-TIC Module 2/mobile/5Wq7xHuw06K_DX928_DY928_CX695_CY688.png]

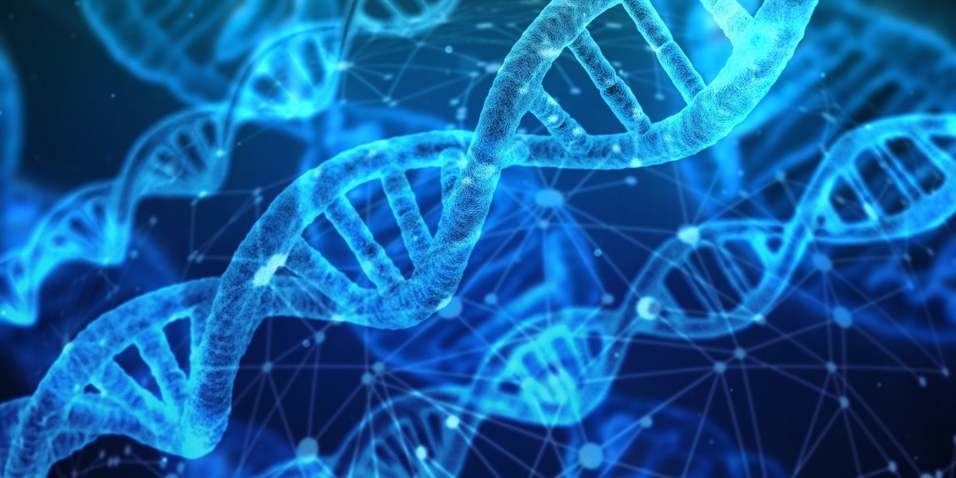

Supplement: Supplementary file 1 — CA-TIC Premodule folderCA-TIC Module 1 folderCA-TIC Module 2 folderCA-TIC Module 3 folderImage Citations.docxCA-TIC Evaluation.docx [file mep_2374-8265.10990-s001.zip › C. CA-TIC Module 2/mobile/5XEWxznToGj_DX1276_DY1276_CX956_CY478.jpg]

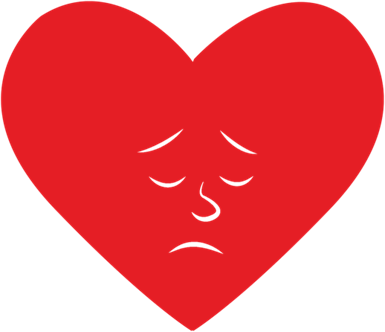

Supplement: Supplementary file 1 — CA-TIC Premodule folderCA-TIC Module 1 folderCA-TIC Module 2 folderCA-TIC Module 3 folderImage Citations.docxCA-TIC Evaluation.docx [file mep_2374-8265.10990-s001.zip › C. CA-TIC Module 2/mobile/5ZJJVFcWvTg_DX514_DY514_CX385_CY332.png]

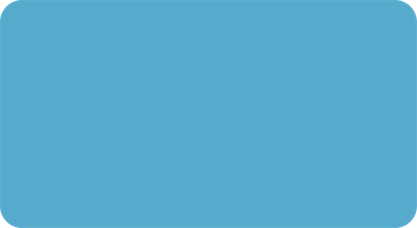

Supplement: Supplementary file 1 — CA-TIC Premodule folderCA-TIC Module 1 folderCA-TIC Module 2 folderCA-TIC Module 3 folderImage Citations.docxCA-TIC Evaluation.docx [file mep_2374-8265.10990-s001.zip › C. CA-TIC Module 2/mobile/67XznMDUUsl_DX604_DY604_CX418_CY228.png]

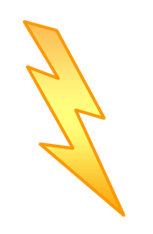

Supplement: Supplementary file 1 — CA-TIC Premodule folderCA-TIC Module 1 folderCA-TIC Module 2 folderCA-TIC Module 3 folderImage Citations.docxCA-TIC Evaluation.docx [file mep_2374-8265.10990-s001.zip › C. CA-TIC Module 2/mobile/68TuGgqZkmq_DX312_DY312_CX154_CY234.png]

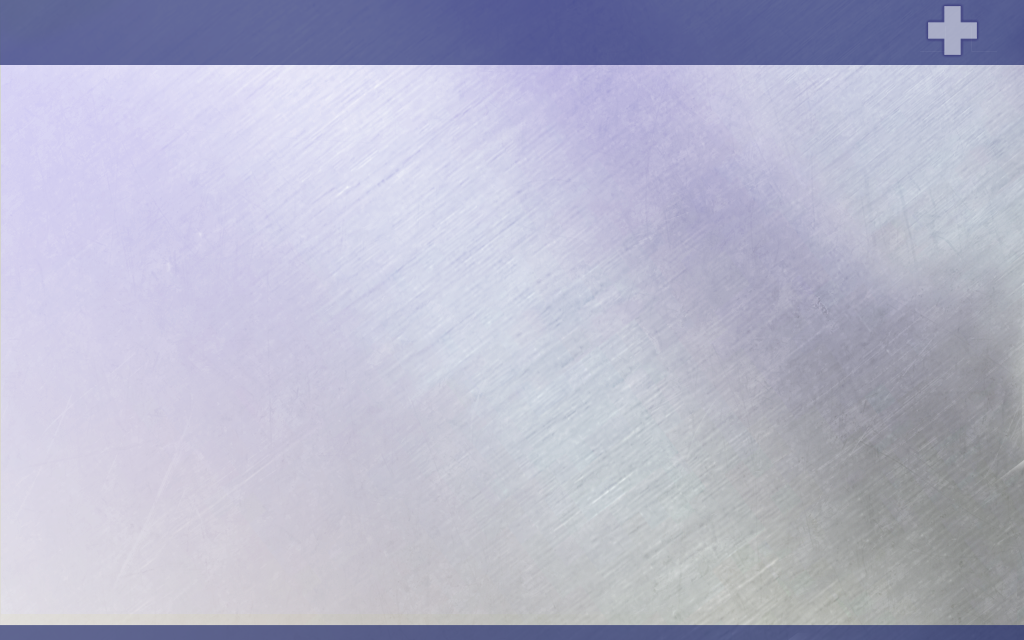

Supplement: Supplementary file 1 — CA-TIC Premodule folderCA-TIC Module 1 folderCA-TIC Module 2 folderCA-TIC Module 3 folderImage Citations.docxCA-TIC Evaluation.docx [file mep_2374-8265.10990-s001.zip › C. CA-TIC Module 2/mobile/6A0Y3Bi3Imx_DX2048_DY2048_CX1024_CY640.png]

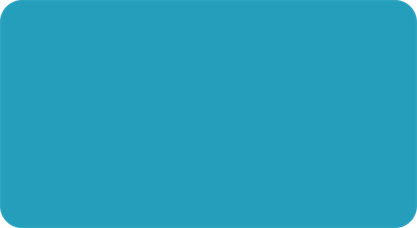

Supplement: Supplementary file 1 — CA-TIC Premodule folderCA-TIC Module 1 folderCA-TIC Module 2 folderCA-TIC Module 3 folderImage Citations.docxCA-TIC Evaluation.docx [file mep_2374-8265.10990-s001.zip › C. CA-TIC Module 2/mobile/6bKMV1YZ35T_DX604_DY604_CX418_CY228.png]

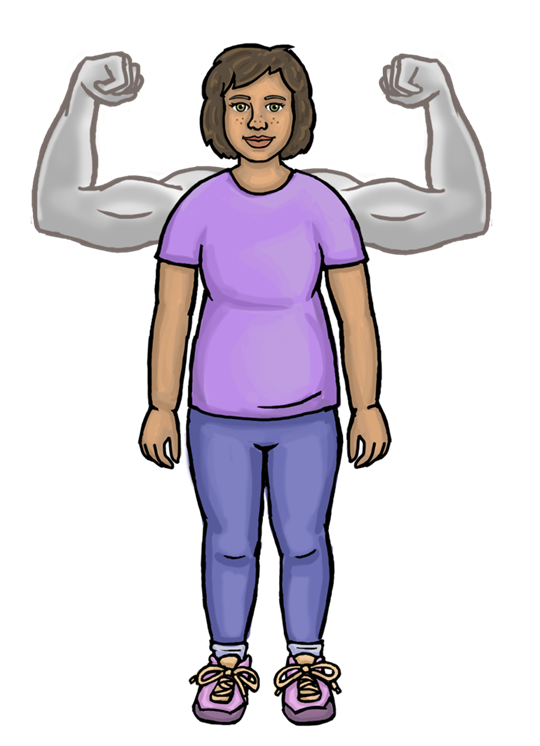

Supplement: Supplementary file 1 — CA-TIC Premodule folderCA-TIC Module 1 folderCA-TIC Module 2 folderCA-TIC Module 3 folderImage Citations.docxCA-TIC Evaluation.docx [file mep_2374-8265.10990-s001.zip › C. CA-TIC Module 2/mobile/6cq3xNRk58k_DX996_DY996_CX540_CY746.png]

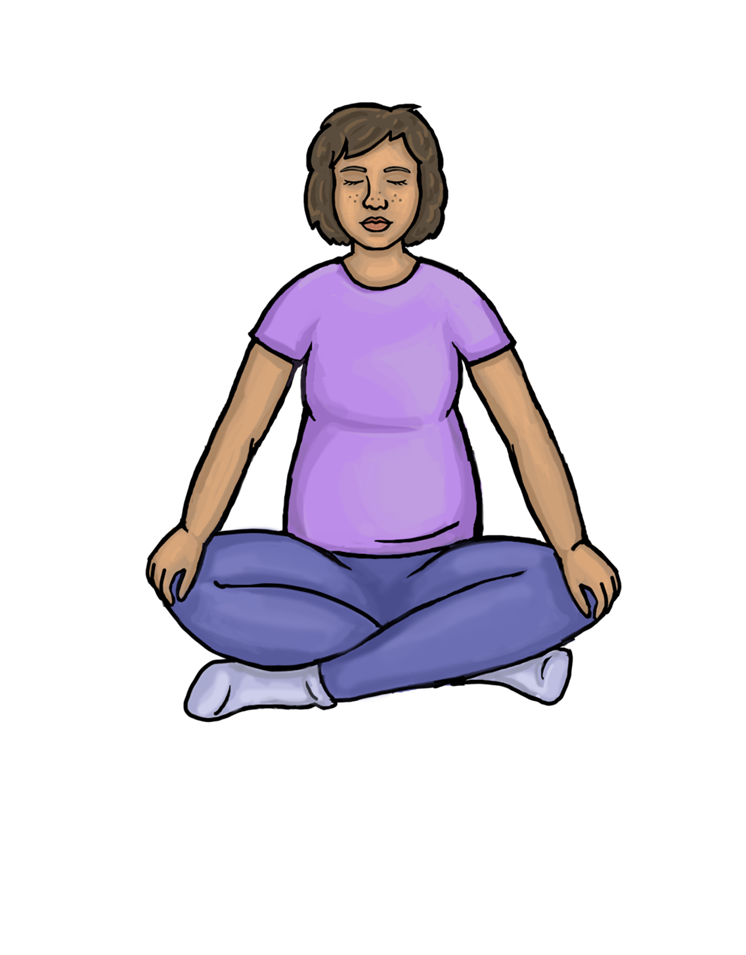

Supplement: Supplementary file 1 — CA-TIC Premodule folderCA-TIC Module 1 folderCA-TIC Module 2 folderCA-TIC Module 3 folderImage Citations.docxCA-TIC Evaluation.docx [file mep_2374-8265.10990-s001.zip › C. CA-TIC Module 2/mobile/6ee7kDwyzE0_DX1274_DY1274_CX746_CY955.png]

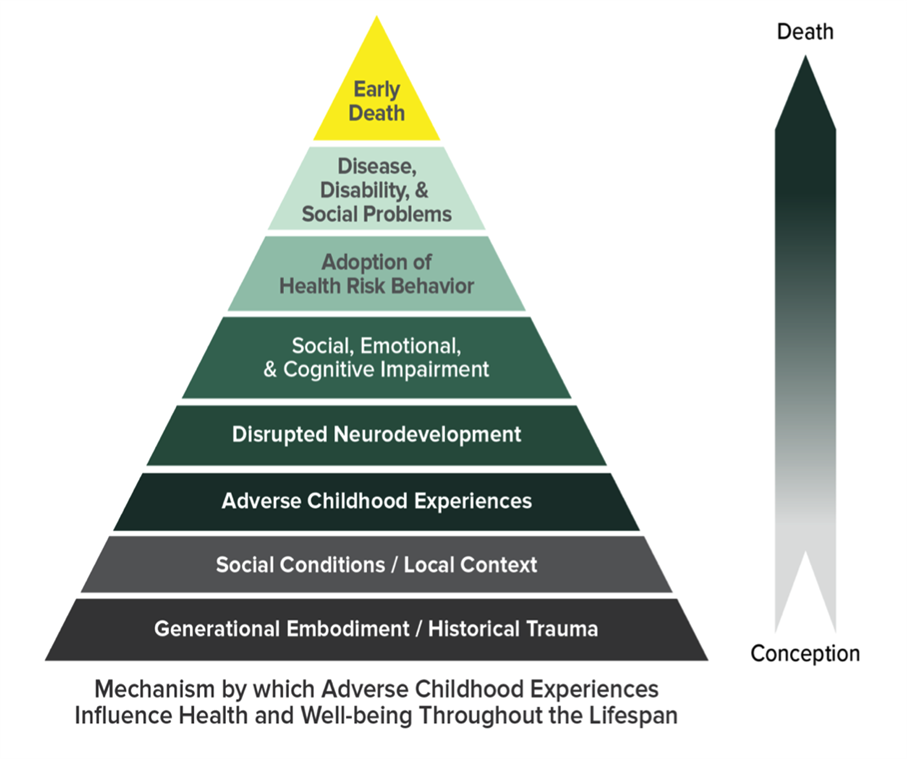

Supplement: Supplementary file 1 — CA-TIC Premodule folderCA-TIC Module 1 folderCA-TIC Module 2 folderCA-TIC Module 3 folderImage Citations.docxCA-TIC Evaluation.docx [file mep_2374-8265.10990-s001.zip › C. CA-TIC Module 2/mobile/6ey6BKiberi_DX1212_DY1212_CX908_CY760.png]

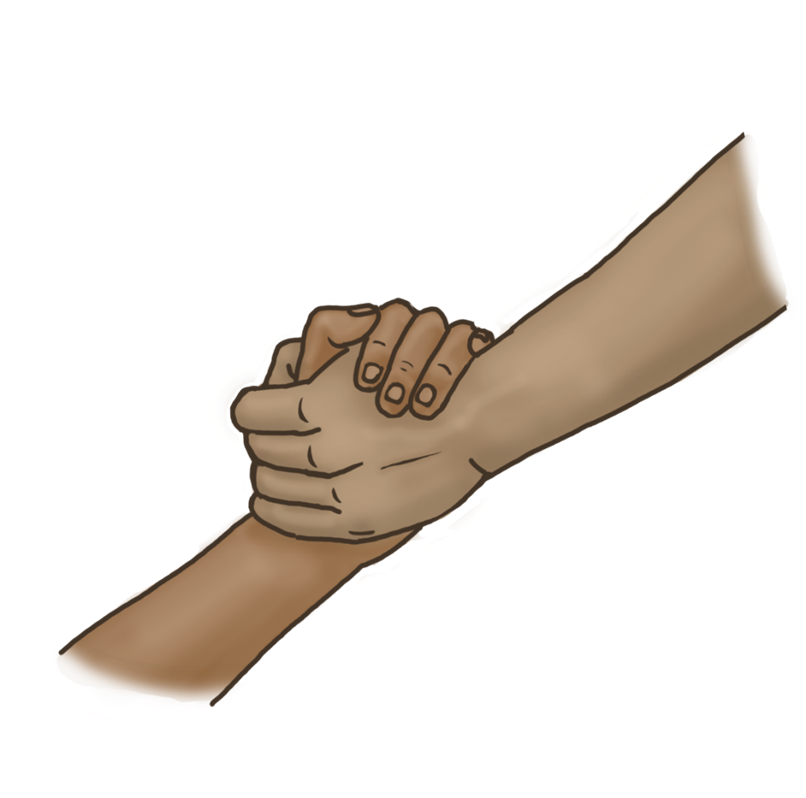

Supplement: Supplementary file 1 — CA-TIC Premodule folderCA-TIC Module 1 folderCA-TIC Module 2 folderCA-TIC Module 3 folderImage Citations.docxCA-TIC Evaluation.docx [file mep_2374-8265.10990-s001.zip › C. CA-TIC Module 2/mobile/6Fo9mAbimgL_DX1076_DY1076_CX807_CY798.png]

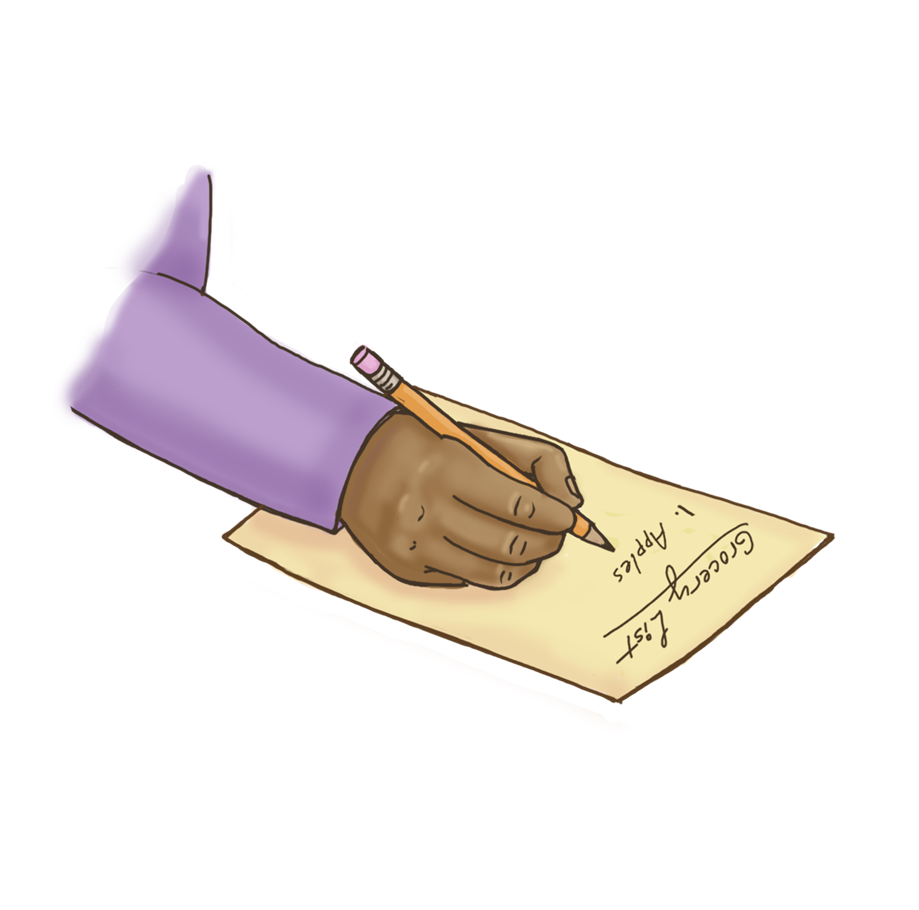

Supplement: Supplementary file 1 — CA-TIC Premodule folderCA-TIC Module 1 folderCA-TIC Module 2 folderCA-TIC Module 3 folderImage Citations.docxCA-TIC Evaluation.docx [file mep_2374-8265.10990-s001.zip › C. CA-TIC Module 2/mobile/6fP7b615RRY_DX1228_DY1228_CX920_CY910.png]

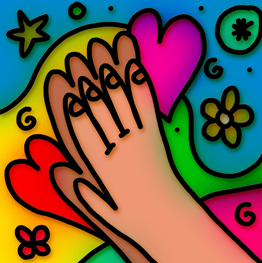

Supplement: Supplementary file 1 — CA-TIC Premodule folderCA-TIC Module 1 folderCA-TIC Module 2 folderCA-TIC Module 3 folderImage Citations.docxCA-TIC Evaluation.docx [file mep_2374-8265.10990-s001.zip › C. CA-TIC Module 2/mobile/6hjssKDd0Oi_DX444_DY444_CX262_CY263.png]

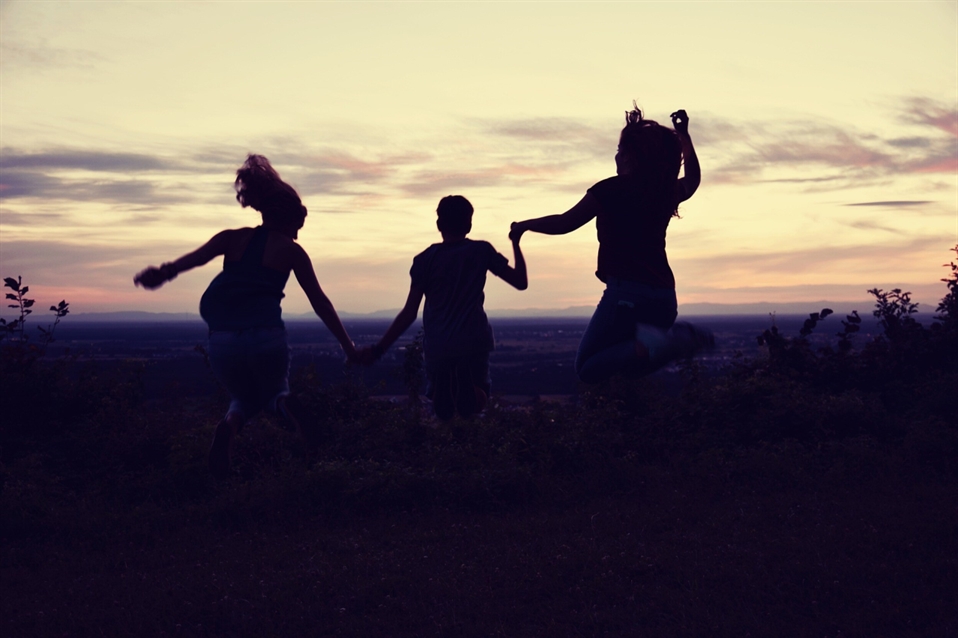

Supplement: Supplementary file 1 — CA-TIC Premodule folderCA-TIC Module 1 folderCA-TIC Module 2 folderCA-TIC Module 3 folderImage Citations.docxCA-TIC Evaluation.docx [file mep_2374-8265.10990-s001.zip › C. CA-TIC Module 2/mobile/6HNgKGYxdMk_DX1278_DY1278_CX958_CY639.jpg]

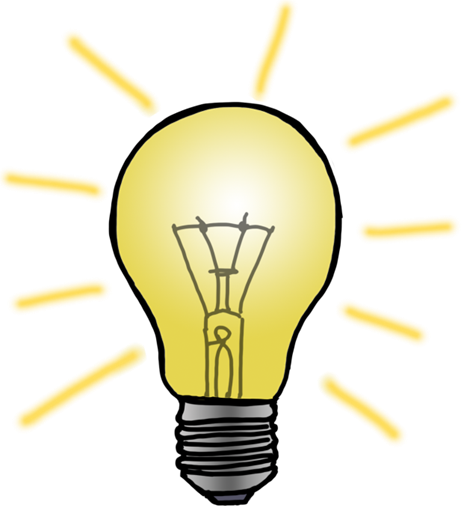

Supplement: Supplementary file 1 — CA-TIC Premodule folderCA-TIC Module 1 folderCA-TIC Module 2 folderCA-TIC Module 3 folderImage Citations.docxCA-TIC Evaluation.docx [file mep_2374-8265.10990-s001.zip › C. CA-TIC Module 2/mobile/6I0Qx96CGKw_DX702_DY702_CX465_CY526.png]

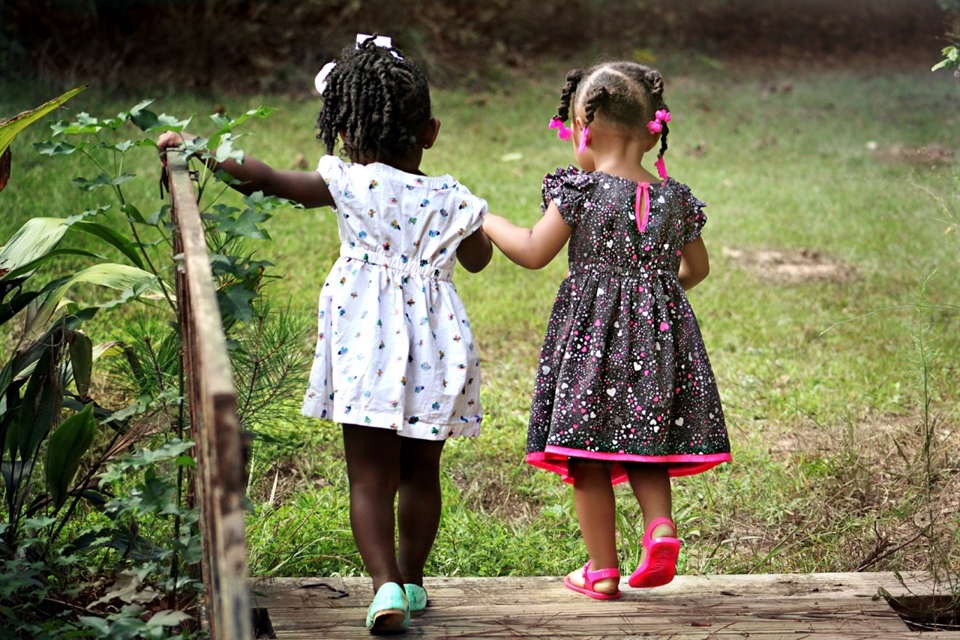

Supplement: Supplementary file 1 — CA-TIC Premodule folderCA-TIC Module 1 folderCA-TIC Module 2 folderCA-TIC Module 3 folderImage Citations.docxCA-TIC Evaluation.docx [file mep_2374-8265.10990-s001.zip › C. CA-TIC Module 2/mobile/6iAAd46nske_DX1280_DY1280_CX960_CY640.jpg]

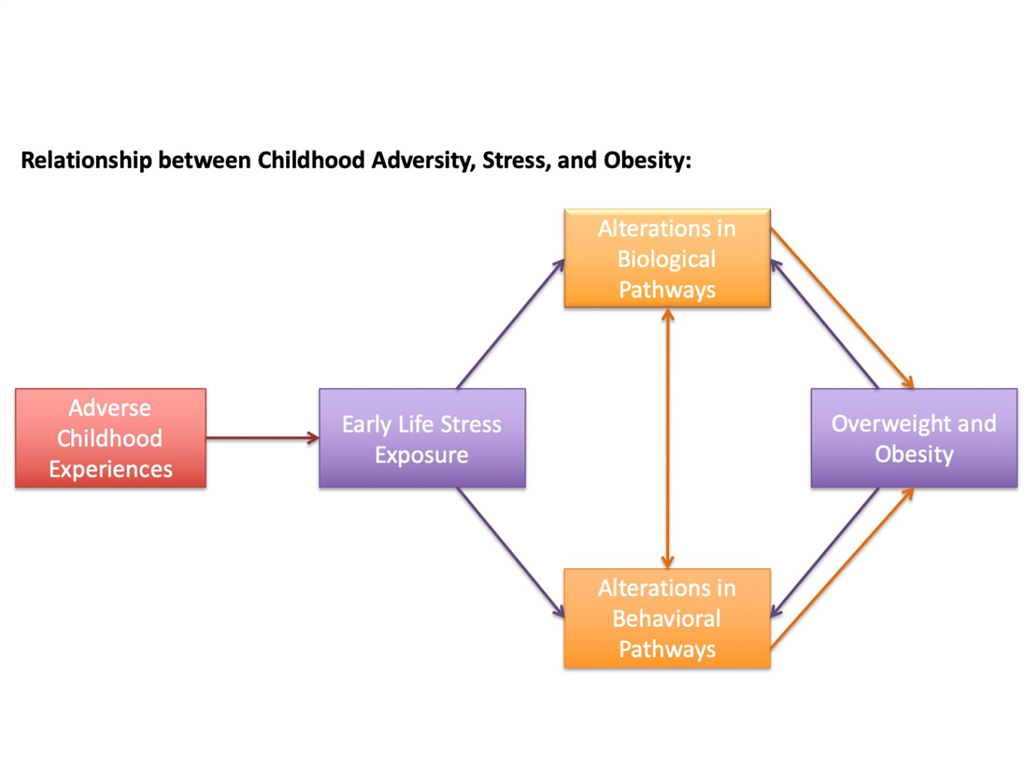

Supplement: Supplementary file 1 — CA-TIC Premodule folderCA-TIC Module 1 folderCA-TIC Module 2 folderCA-TIC Module 3 folderImage Citations.docxCA-TIC Evaluation.docx [file mep_2374-8265.10990-s001.zip › C. CA-TIC Module 2/mobile/6JCknVvvu6h_DX1368_DY1368_CX1025_CY769.png]

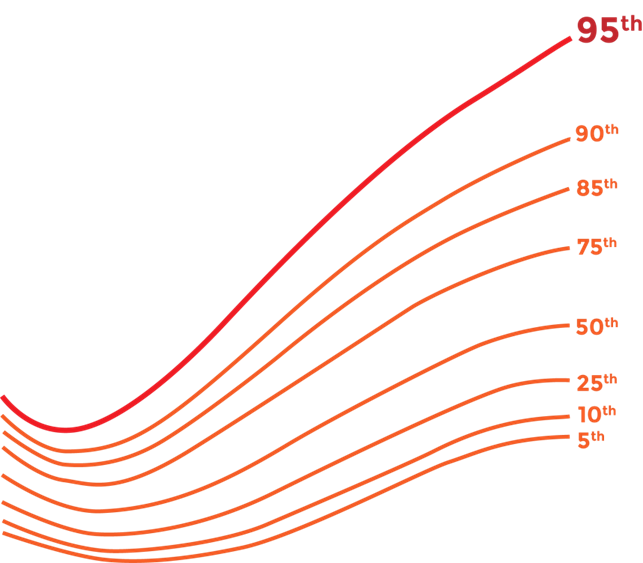

Supplement: Supplementary file 1 — CA-TIC Premodule folderCA-TIC Module 1 folderCA-TIC Module 2 folderCA-TIC Module 3 folderImage Citations.docxCA-TIC Evaluation.docx [file mep_2374-8265.10990-s001.zip › C. CA-TIC Module 2/mobile/6K3CuyFWt3W_DX858_DY858_CX643_CY563.png]

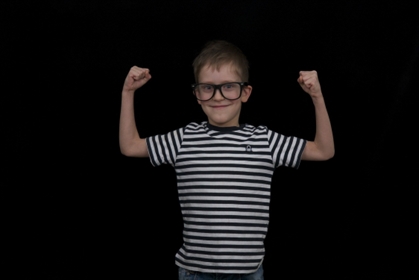

Supplement: Supplementary file 1 — CA-TIC Premodule folderCA-TIC Module 1 folderCA-TIC Module 2 folderCA-TIC Module 3 folderImage Citations.docxCA-TIC Evaluation.docx [file mep_2374-8265.10990-s001.zip › C. CA-TIC Module 2/mobile/6LW2MZSumxi_DX562_DY562_CX420_CY280.jpg]

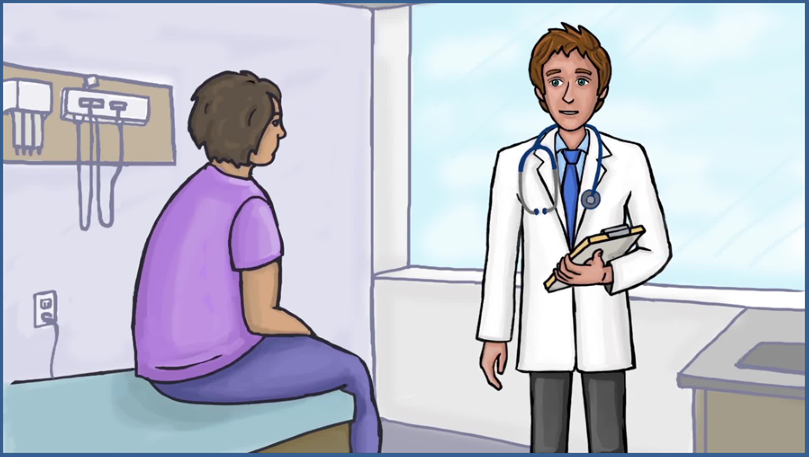

Supplement: Supplementary file 1 — CA-TIC Premodule folderCA-TIC Module 1 folderCA-TIC Module 2 folderCA-TIC Module 3 folderImage Citations.docxCA-TIC Evaluation.docx [file mep_2374-8265.10990-s001.zip › C. CA-TIC Module 2/mobile/6mMv2K7Oepj_DX1618_DY1618_CX809_CY457.png]

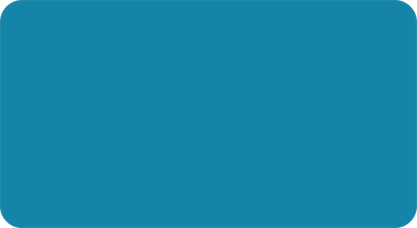

Supplement: Supplementary file 1 — CA-TIC Premodule folderCA-TIC Module 1 folderCA-TIC Module 2 folderCA-TIC Module 3 folderImage Citations.docxCA-TIC Evaluation.docx [file mep_2374-8265.10990-s001.zip › C. CA-TIC Module 2/mobile/6MpK04DyJzT_DX604_DY604_CX418_CY228.png]

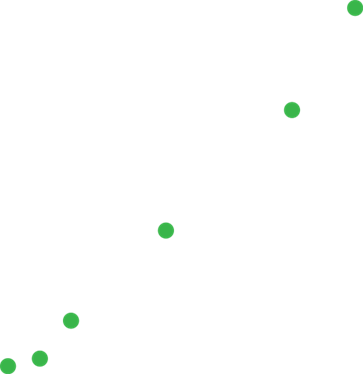

Supplement: Supplementary file 1 — CA-TIC Premodule folderCA-TIC Module 1 folderCA-TIC Module 2 folderCA-TIC Module 3 folderImage Citations.docxCA-TIC Evaluation.docx [file mep_2374-8265.10990-s001.zip › C. CA-TIC Module 2/mobile/6mvpEtR7QEr_DX500_DY500_CX363_CY375.png]

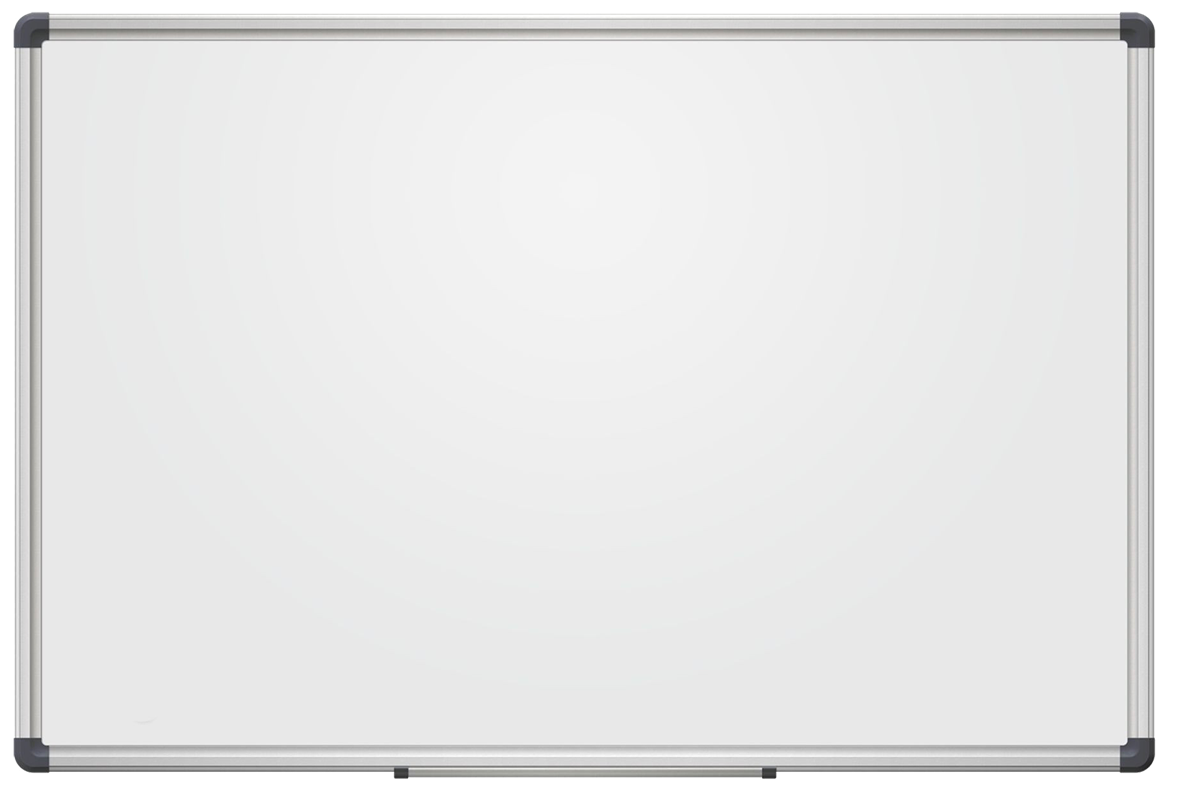

Supplement: Supplementary file 1 — CA-TIC Premodule folderCA-TIC Module 1 folderCA-TIC Module 2 folderCA-TIC Module 3 folderImage Citations.docxCA-TIC Evaluation.docx [file mep_2374-8265.10990-s001.zip › C. CA-TIC Module 2/mobile/6nAYD602p6V_DX1760_DY1760_CX1177_CY793.png]

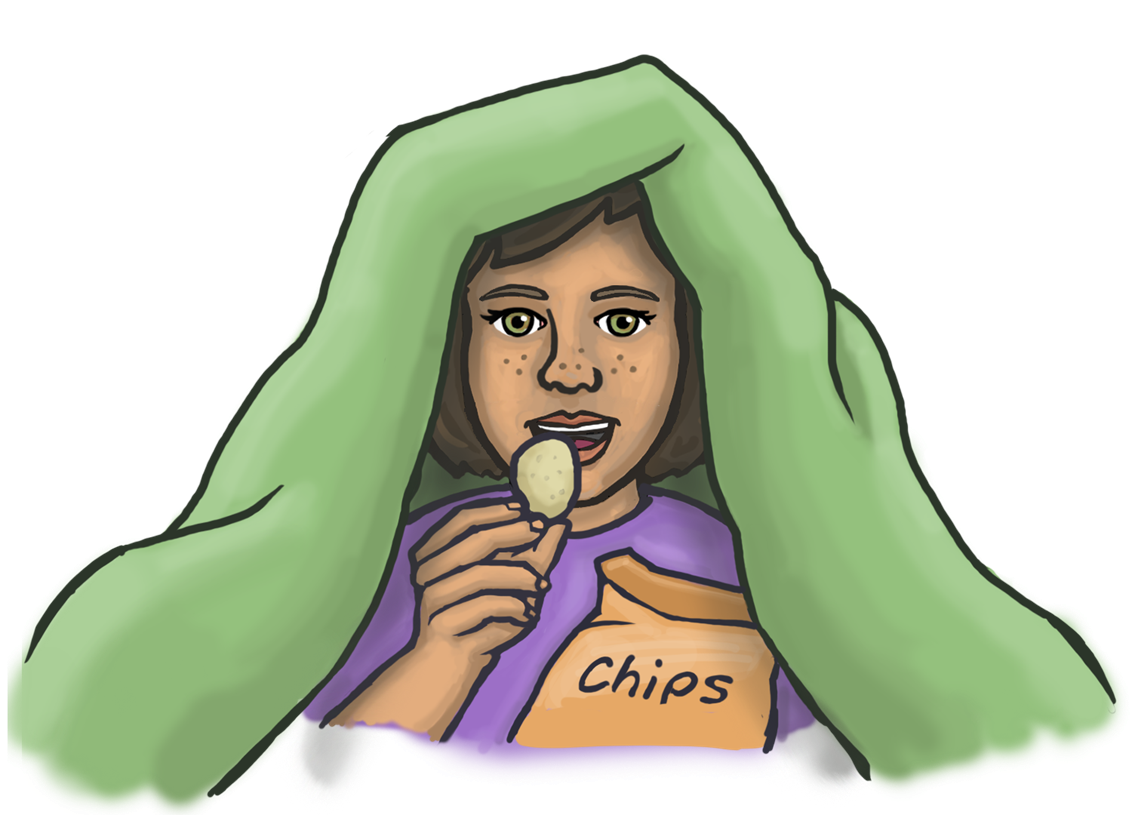

Supplement: Supplementary file 1 — CA-TIC Premodule folderCA-TIC Module 1 folderCA-TIC Module 2 folderCA-TIC Module 3 folderImage Citations.docxCA-TIC Evaluation.docx [file mep_2374-8265.10990-s001.zip › C. CA-TIC Module 2/mobile/6nQ5O1NAtqT_DX1510_DY1510_CX1132_CY815.png]

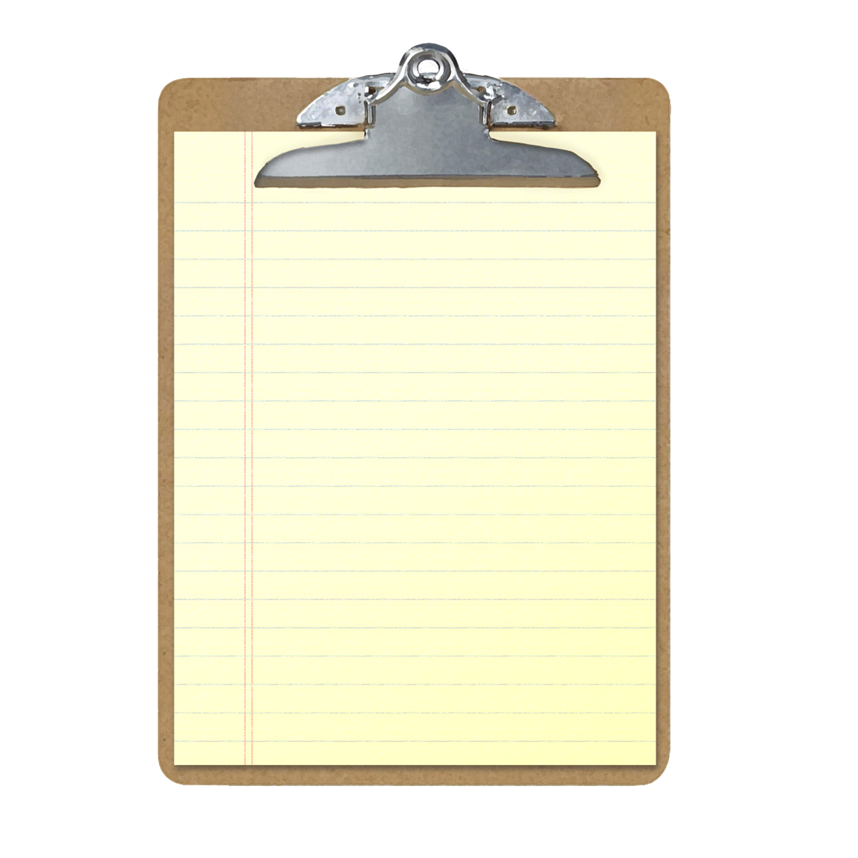

Supplement: Supplementary file 1 — CA-TIC Premodule folderCA-TIC Module 1 folderCA-TIC Module 2 folderCA-TIC Module 3 folderImage Citations.docxCA-TIC Evaluation.docx [file mep_2374-8265.10990-s001.zip › C. CA-TIC Module 2/mobile/6nTykGQrWxU_DX1144_DY1144_CX858_CY858.png]

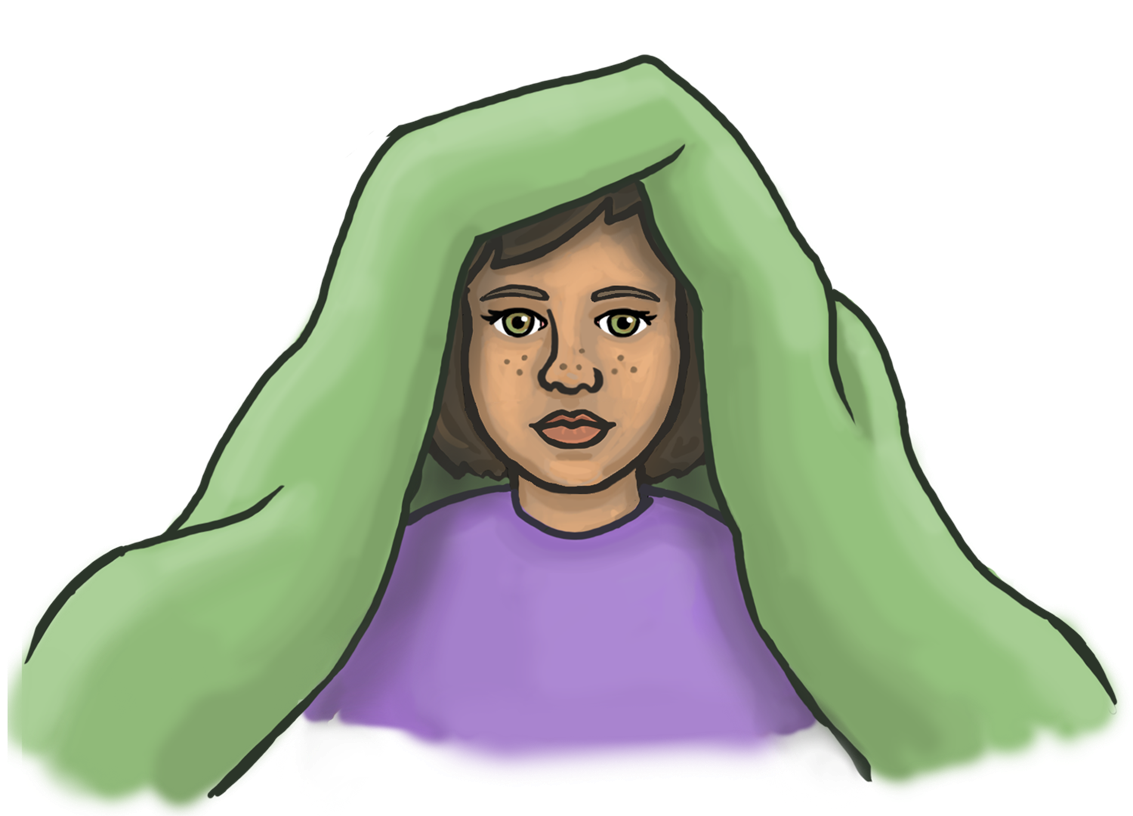

Supplement: Supplementary file 1 — CA-TIC Premodule folderCA-TIC Module 1 folderCA-TIC Module 2 folderCA-TIC Module 3 folderImage Citations.docxCA-TIC Evaluation.docx [file mep_2374-8265.10990-s001.zip › C. CA-TIC Module 2/mobile/6nyXmTPmr9n_DX1512_DY1512_CX1133_CY816.png]

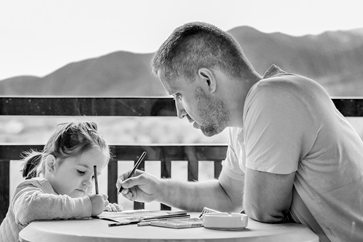

Supplement: Supplementary file 1 — CA-TIC Premodule folderCA-TIC Module 1 folderCA-TIC Module 2 folderCA-TIC Module 3 folderImage Citations.docxCA-TIC Evaluation.docx [file mep_2374-8265.10990-s001.zip › C. CA-TIC Module 2/mobile/6SGXMJAeWRp_DX712_DY712_CX363_CY242.png]

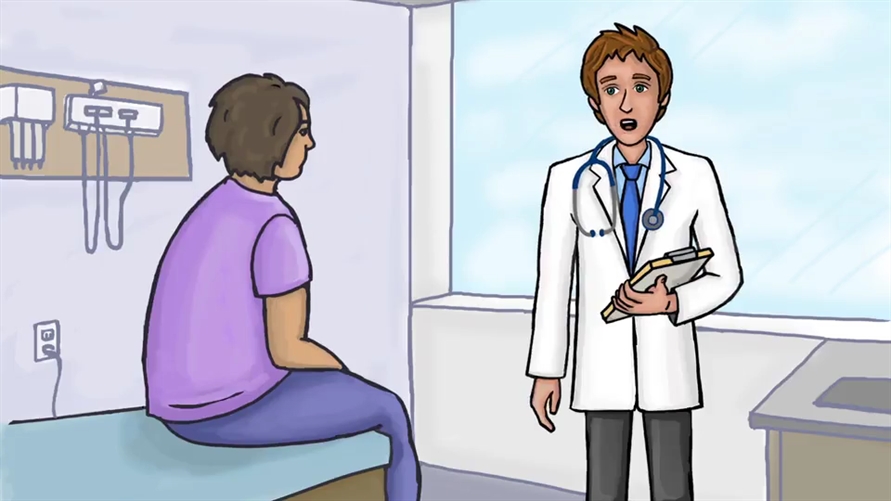

Supplement: Supplementary file 1 — CA-TIC Premodule folderCA-TIC Module 1 folderCA-TIC Module 2 folderCA-TIC Module 3 folderImage Citations.docxCA-TIC Evaluation.docx [file mep_2374-8265.10990-s001.zip › C. CA-TIC Module 2/mobile/poster_6LuwOj64645_video_6rnYrDrzhK9_18_48_892x502.jpg]

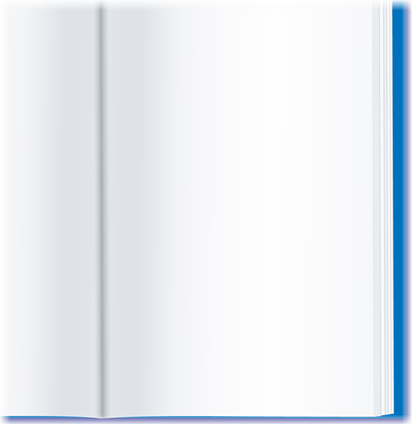

Supplement: Supplementary file 1 — CA-TIC Premodule folderCA-TIC Module 1 folderCA-TIC Module 2 folderCA-TIC Module 3 folderImage Citations.docxCA-TIC Evaluation.docx [file mep_2374-8265.10990-s001.zip › C. CA-TIC Module 2/mobile/Shape67IpnisQNM9.png]

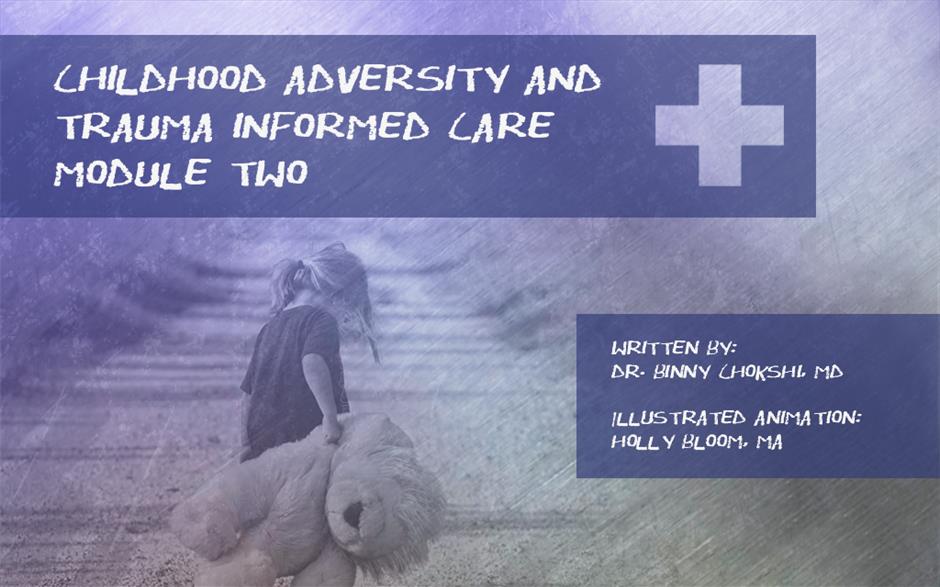

Supplement: Supplementary file 1 — CA-TIC Premodule folderCA-TIC Module 1 folderCA-TIC Module 2 folderCA-TIC Module 3 folderImage Citations.docxCA-TIC Evaluation.docx [file mep_2374-8265.10990-s001.zip › C. CA-TIC Module 2/story_content/thumbnail.jpg]

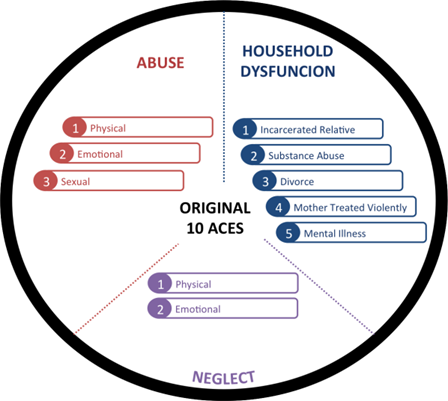

Supplement: Supplementary file 1 — CA-TIC Premodule folderCA-TIC Module 1 folderCA-TIC Module 2 folderCA-TIC Module 3 folderImage Citations.docxCA-TIC Evaluation.docx [file mep_2374-8265.10990-s001.zip › D. CA-TIC Module 3/mobile/5cA65Wd64gi_DX598_DY598_CX448_CY402.png]

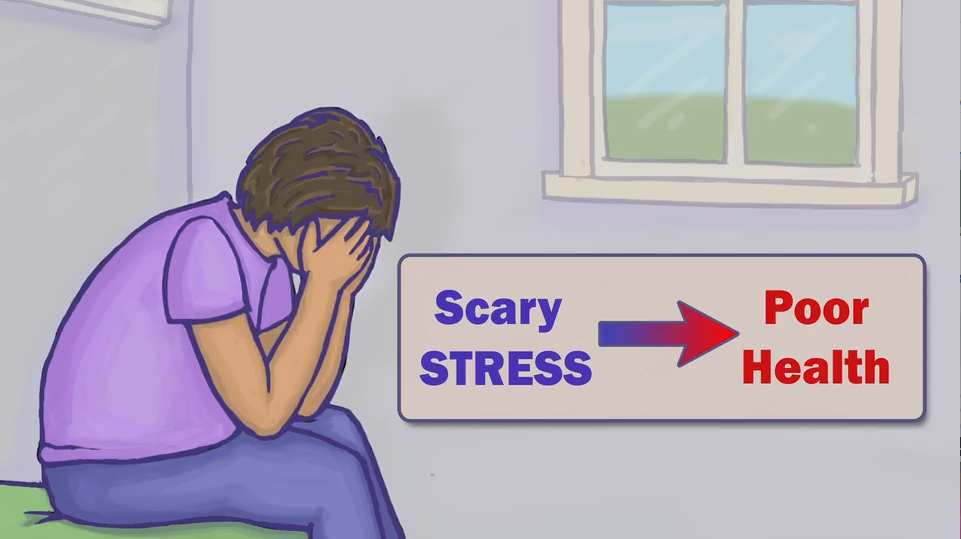

Supplement: Supplementary file 1 — CA-TIC Premodule folderCA-TIC Module 1 folderCA-TIC Module 2 folderCA-TIC Module 3 folderImage Citations.docxCA-TIC Evaluation.docx [file mep_2374-8265.10990-s001.zip › D. CA-TIC Module 3/mobile/5cuSCjrToIG_DX1556_DY1556_CX961_CY539.png]

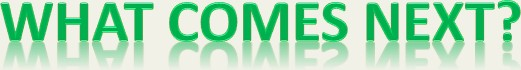

Supplement: Supplementary file 1 — CA-TIC Premodule folderCA-TIC Module 1 folderCA-TIC Module 2 folderCA-TIC Module 3 folderImage Citations.docxCA-TIC Evaluation.docx [file mep_2374-8265.10990-s001.zip › D. CA-TIC Module 3/mobile/5j2EFi1QGXE_DX1042_DY1042_CX521_CY70.png]

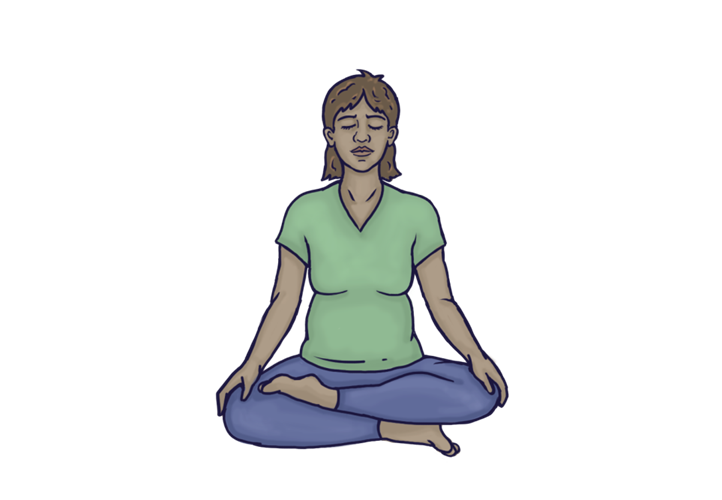

Supplement: Supplementary file 1 — CA-TIC Premodule folderCA-TIC Module 1 folderCA-TIC Module 2 folderCA-TIC Module 3 folderImage Citations.docxCA-TIC Evaluation.docx [file mep_2374-8265.10990-s001.zip › D. CA-TIC Module 3/mobile/5lwnFGzqyEL_DX964_DY964_CX723_CY501.png]
