# Supplementary material for: Interactive Case-Based Childhood Adversity and Trauma-Informed Care Electronic Modules for Pediatric Primary Care
Source: MedEdPORTAL. 2020 Oct 12;16:10990. doi: 10.15766/mep_2374-8265.10990 (PMC7549390; doi:10.15766/mep_2374-8265.10990)
Supplement: Supplementary file 1 — CA-TIC Premodule folderCA-TIC Module 1 folderCA-TIC Module 2 folderCA-TIC Module 3 folderImage Citations.docxCA-TIC Evaluation.docx [file mep_2374-8265.10990-s001.zip › E. Image Citations.docx]

MEP-2019-0393 Appendix E: CA-TIC Module Image Citations

Title: Interactive Case Based Childhood Adversity and Trauma Informed Care Electronic Modules for Pediatric Primary Care

Corresponding Author: Binny Chokshi, MD (bchokshi@cnmc.org)

**Pre Module**

**Slide 3:**

**10 ACES:**

Image created by Ruchi Parikh, used with permission.

**Slide 4**

**Early Adversity Health Effects:**

Image by Centers for Disease Control and Prevention, retrieved from https://www.cdc.gov/violenceprevention/childabuseandneglect/acestudy/ace-graphics.html on December 21, 2019. Image is in the public domain.

**Slide 8**

**ACE pyramid:**

Image by Centers for Disease Control and Prevention, retrieved from https://www.cdc.gov/violenceprevention/childabuseandneglect/acestudy/ace-graphics.html on December 21, 2019. Image is in the public domain.

**Slide 9**

**Swing**

Image by biancamentil, retrieved from https://pixabay.com/photos/swing-playground-swing-device-1218654/ on December 6, 2019. Image is in the public domain.

**Slide 10**

**Social Determinants of Health:**

Image created on word- by Binny Chokshi

**Module 1:**

**1.1: Title Slide:**

Girl with Bear:

Image by greyerbaby, retrieved from https://pixabay.com/photos/girl-walking-teddy-bear-child-walk-447701/ on September 19, 2017. Image is in the public domain.

**1.2 Introduction**

Binny Avatar:

Image created by Holly Bloom, used with permission.

**1.3 Adverse Childhood Experiences:**

10 Adverse Childhood Experiences:

**I**mage created by Ruchi Parikh, used with permission

**1.4 Social Determinants of Health:**

Circle image:

Image created by Binny Chokshi

National Survey of Children’s Health Data Image:

Created by Binny Chokshi

**1.5 Principles of Trauma informed care:**

a.) Girl with scary🡪stress: Image created by Holly Bloom, used with permission

b.) Computer with stressors: image created by Holly Bloom, used with permission

c.) Psychologist with patient: image created by Holly Bloom, used with permission

d.) 1.) Hands / Pulse / Heart : Image by geralt, retrieved from <https://pixabay.com/illustrations/heart-health-pulse-heart-rate-1616463/> , on September 19, 2017. Image is in the public domain.

**1.6 Principles of Trauma Informed Care 2:**

Doctor:

Image created by Holly Bloom, used with permission.

**1.7 Tenet of Trauma Informed Care:**

Picture of lake/mountains/clouds:

Image by pixabay, retrieved from [**https://pixabay.com/photos/hintersee-bergsee-mountains-ramsau-3601004/**](https://pixabay.com/photos/hintersee-bergsee-mountains-ramsau-3601004/), on December 9^th^, 2019. Image is in the public domain.

**1.8 Case 1:**

Kevin:

Image created by Holly Bloom, used with permission.

**1.9 Case 1 Continued:**

Doctor/Kevin/Mother:

Image created by Holly Bloom, used with permission.

**1.17 Kevin Experiences Trauma:**

Police Line Do Not Cross:

Image by geralt, retrieved from <https://pixabay.com/illustrations/police-crime-scene-blue-light-862341/> on September 19, 2017. Image is in the public domain.

**1.19 Case revisited:**

Kevin:

Image created by Holly Bloom, used with permission.

**1.20 Case review:**

a.) Doctor/Kevin/Mother: Image created by Holly Bloom, used with permission.

b.) Bed: Image created by Holly Bloom, used with permission.

c.) Rx Bottle: Image created by Holly Bloom, used with permission.

d.) Stress Clipboard: Image created by Holly Bloom, used with permission.

e.) Health Serpent: Image created by Holly Bloom, used with permission.

f.) Police Line Do Not cross: Image created by Holly Bloom, used with permission.

g.) Psychologist: Image created by Holly Bloom, used with permission

**1.21 Clinical Pearl:**

Image by National Child Traumatic Stress Network. Retrieved from <https://www.nctsn.org/sites/default/files/resources//is_it_adhd_or_child_traumatic_stress.pdf>

on September 2017. NCTSN is part of Substance Abuse and Mental Health Services Administration, federal agency, therefore image is in the public domain.

**Module 2:**

**1.1 Title Slide:**

Girl with Bear:

Image by greyerbaby, retrieved from https://pixabay.com/photos/girl-walking-teddy-bear-child-walk-447701/ on September 19, 2017. Image is in the public domain.

**1.2 What is Trauma Informed Care:**

ACE Pyramid:

Image by Centers for Disease Control and Prevention, retrieved from https://www.cdc.gov/violenceprevention/childabuseandneglect/acestudy/ace-graphics.html on December 21, 2019. Image is in the public domain.

**1.3 Resilience 1:**

a.) Girls holding hands: Image by cherylholt, retrieved from <https://pixabay.com/photos/girls-children-kids-friends-young-462072/> on December 9, 2019. Image is in the public domain
b.) DNA Helix: Image by geralt, retrieved from <https://pixabay.com/illustrations/dna-genetic-material-helix-proteins-3539309/> on December 9, 2019. Image is in the public domain.

**1.4 Resilience 2:**

Resilience Pathway:

Image created by Binny Chokshi

**1.5 Resilience 3:**

a.) Girl Father Portrait: Image by ddimitrova, retrieved from https://pixabay.com/photos/girl-father-portrait-eyes-people-1641215/ on September 19, 2017. Image is in the public domain.

b.) Kid making muscle: Image by elementus, retrived from <https://pixabay.com/photos/kid-boy-glasses-fun-joy-388527/> on December 9, 2019. Image is in the public domain.

c.) Girl thinking: Image by 5D media, image retrieved from <https://pixabay.com/photos/thinking-kyliebear-kyliebear323-3484737/> on December 9, 2019. Image is in the public domain.

d.) Hand/Heart: Image by Prawny, retrieved from <https://pixabay.com/illustrations/modern-art-hands-team-together-1127824/>, on December 9, 2019. Image is in the public domain.

**1.6 Resilience 4:**

Book:

Image created by Holly Bloom, Used with Permission

**1.7 Chart:**

a.) Patient: Image created by Holly Bloom, used with permission, b.) BMI Chart: Image created by Holly Bloom, used with permission.

**1.9 Trauma Informed Care Principles:**

Patient/Doctor:

Image created by Holly Bloom, used with permission

**1.10 Case 2:**

a.) Patient/Doctor: Image created by Holly Bloom, used with permission
b.) Last year talking heads: Image created by Holly Bloom, used with permission,
c.) Skeleton/organ systems: Image created by Holly Bloom, used with permission,
d.) Chalkboard: Image created by Holly Bloom, used with permission
e.) Calendar: Image created by Holly Bloom, used with permission,
f.) Salad bowl: Image created by Holly Bloom, used with permission,
g.) Scale: Image created by Holly Bloom, used with permission,
h.) Pencil breaking: Image created by Holly Bloom, used with permission
i.) Monster: Image created by Holly Bloom, used with permission,
j.) Bipolar: image created by Holly Bloom, used with permission,
k.) House/hand: Image created by Holly Bloom, used with permission,
l.) Girl/chips: Image created by Holly Bloom, used with permission

**1.12 Case 2 Revisited:**

a.) Clipboard: Image created by Holly Bloom, used with permission.
b.) Lightbulb: Image created by Holly Bloom, used with permission
c.) Patient/Muscles: Image created by Holly Bloom, used with permission
d.) Hand: Image created by Holly Bloom, used with permission
e.) Health Serpent: Image created by Holly Bloom, used with permission
f.) Hands shaking: Image created by Holly Bloom, used with permission
g.) Not so much/honest: Image created by Holly Bloom, used with permission
h.) Hand/writing: Image created by Holly Bloom, used with permission
i.) Girl meditating: Image created by Holly Bloom, used with permission

**1.13 Resilience in Summary:**

Family/Sunset: Image by SimsalabimSabrina, retrieved from [https://pixabay.com/photos/family-sunset-friendship-friends-2609528/](https://pixabay.com/photos/family-sunset-friendship-friends-2609528/on) on December 9, 2019. Image is in the public domain.

**1.14 Summary:**

a.) Patient/doctor: Image created by Holly Bloom, used with permission
b.) Brain: Image created by Holly Bloom, used with permission
c.) Heart: Image by Vishnu_KV, retrieved from <https://pixabay.com/illustrations/crying-heart-heart-cry-love-2318164/>, image is in the public domain.
d.) Family Gathering: Image by mohamed_hassan, retrieved from https://pixabay.com/illustrations/family-gathering-grandparents-father-3068994/on September 19, 2017. Image is in the public domain.

**1.15 Clinical Pearl:**

a.) 10 ACES: Image created by Ruchi Parikh, used with permission
b.) Stress/Obesity Chart: adapted from Miller AL, Dawson L, Welker E. Early Life Stress and Childhood Obesity Risk. <http://www.healthyeatingresearch.org>. Published June 2017. Accessed February 9, 2018.
c.) Girl with chips: Image created by Holly Bloom, used with permission.

**Module 3:**

**1.1 Title Slide:**

Girl with Bear:

Image by greyerbaby, retrieved from https://pixabay.com/photos/girl-walking-teddy-bear-child-walk-447701/ on September 19, 2017. Image is in the public domain.

**1.4 Practical Considerations**

a.) Man with chart: Image created by Holly Bloom, used with permission
b.) Doctor listening to heart: image created by Holly Bloom used with permission
c.) Woman meditating: image created by Holly Bloom used with permission.
d.) 10 ACEs image: Image created by Ruchi Parikh, used with permission
d.) Social Determinants of Health image: Image created in word, by Binny Chokshi
e.) Clipboard: image created by holly bloom, used with permission
f.) Three people: image created by Holly Bloom, used with permission
g.) Computer: image created by holly bloom, used with permission
h.) Pencil/paper: image created by holly bloom, used with permission
i.) Translate: Image created by geralt, retrieved from https://pixabay.com/illustrations/globe-world-languages-translate-110775/ on December 9, 2019. Image is in the public domain.

**1.5 Introducing an ACE Questionnaire**

Doctor/Patient/Mother

Image created by Holly Bloom used with permission

**1.6 After Disclosure of Adversity**

Doctor/Patient/Mother

Image created by Holly Bloom used with permission

**1.7 Education**

Doctor/Patient/Mother

Image created by Holly Bloom used with permission

**1.8 Resources:**

a.) Patient/Mother/Woman with Community Resources: Images by Holly Bloom, used with permission
b.) Mom/Dad/Son: image is by badski007, retrieved from <https://pixabay.com/photos/family-people-child-smile-2811003/> on December 9, 2019. Image is in the public domain.
c.) Salad bowl: Image created by Holly Bloom, used with permission
d.) Kids on bikes: Image by Rhythm_in_life, retreviewed from <https://pixabay.com/photos/bikes-kids-children-summer-2379215/> on December 9, 2019. Image is in the public domain.
e.) Rainbow Hands: Image by Westfrisco, retrieved from https://pixabay.com/illustrations/group-therapy-counseling-health-2351896/ on September 19, 2017. Image is in the public domain
f.) Computer with stressors: Images by Holly Bloom, used with permission
g.) IPAD Health stats: image by mcmurryjulie, retrieved from <https://pixabay.com/vectors/ehr-emr-electronic-medical-record-1476525/> on December 9, 2019. Image is in the public domain
j.) Handshake: Image by shutterbug75, retrieved from https://pixabay.com/photos/agree-agreement-asian-black-1238964/ on September 19, 2017. Image is in the public domain.

**1.9 Universal Precautions:**

Doctor/Patient/Mother:

Images by Holly Bloom, used with permission

Exclamation Point:

Image by Clker-Free-Vector Images, retrieved from: <https://pixabay.com/vectors/exclamation-mark-point-triangle-24144/>. Accessed on April 15, 2020. Image is in the public domain.

**1.12 Module Summary:**

a.) Doctor: image by Holly Bloom, used with permission
b.) Girl, scary🡪stress: image by holly bloom, used with permission
k.) Globe, Kids: Image by pixabay, retrieved from https://pixabay.com/illustrations/people-life-lifestyle-cartoon-kids-2129789/ on September 19, 2017. Image is in the public domain.
