# Supplementary material for: Interactive Case-Based Childhood Adversity and Trauma-Informed Care Electronic Modules for Pediatric Primary Care
Source: MedEdPORTAL. 2020 Oct 12;16:10990. doi: 10.15766/mep_2374-8265.10990 (PMC7549390; doi:10.15766/mep_2374-8265.10990)
Supplement: Supplementary file 1 — CA-TIC Premodule folderCA-TIC Module 1 folderCA-TIC Module 2 folderCA-TIC Module 3 folderImage Citations.docxCA-TIC Evaluation.docx [file mep_2374-8265.10990-s001.zip › F. CA-TIC Evaluation.docx]

Appendix F: CA-TIC Module Evaluation

**PRE TEST:**

Demographics:

1.) What is your training level?
    a. Medical Student
    b. Resident Physician
    c. Fellow Physician°
    d. Attending Physician
2.) What is your primary site of clinical practice?
    a. Private clinic
    b. Academic Center
    c. Federally qualified health center
    d. School based health center
    e. Other (Please specify below)
3.) Your primary site of clinical practice if you selected “other” above:

Knowledge:

1.) Please use the scale to rate your general knowledge on **Adverse Childhood Experiences:**

1= Very Weak 5 = Very strong

| [ ] | [ ] | [ ] | [ ] | [ ] |
| --- | --- | --- | --- | --- |
| 1 | 2 | 3 | 4 | 5 |

2.) Please use the scale to rate your general knowledge on the **link between traumatic exposures and poor health outcomes:**

1= Very Weak 5 = Very strong

| [ ] | [ ] | [ ] | [ ] | [ ] |
| --- | --- | --- | --- | --- |
| 1 | 2 | 3 | 4 | 5 |

3.) Please use the scale below to rate your general knowledge on **the central tenet of trauma informed care:**

1= Very Weak 5 = Very strong

| [ ] | [ ] | [ ] | [ ] | [ ] |
| --- | --- | --- | --- | --- |
| 1 | 2 | 3 | 4 | 5 |

4.) Please use the scale below to rate your general knowledge on **the six principles of trauma informed care:**

1= Very Weak 5 = Very strong

| [ ] | [ ] | [ ] | [ ] | [ ] |
| --- | --- | --- | --- | --- |
| 1 | 2 | 3 | 4 | 5 |

5.) Please use the scale below to rate your general knowledge on **the components of childhood resilience:**

Attitudes:

6.) Please use the screen below to rate how important you **believe screening for traumatic exposures during routine patient visits in the pediatric medical home setting** to be:

1= Not at all important 5 = Very important

| [ ] | [ ] | [ ] | [ ] | [ ] |
| --- | --- | --- | --- | --- |
| 1 | 2 | 3 | 4 | 5 |

7.) Please use the screen below to rate how important you believe **implementation of clinic protocols to standardize screening for and addressing a history of traumatic exposures in patients and caregivers in the pediatric medical home setting** to be:

1= Not at all important 5 = Very important

| [ ] | [ ] | [ ] | [ ] | [ ] |
| --- | --- | --- | --- | --- |
| 1 | 2 | 3 | 4 | 5 |

8.) Please use the scale below to rate how important you believe **discussion with patients and caregivers regarding the relationship between a history of trauma exposure and poor health outcomes** to be:

1= Not at all important 5 = Very important

| [ ] | [ ] | [ ] | [ ] | [ ] |
| --- | --- | --- | --- | --- |
| 1 | 2 | 3 | 4 | 5 |

9.) Please use the scale below to rate how important you believe **accessible training opportunities regarding the relationship between trauma and health and the principles of trauma informed care for all staff working within the pediatric medical home setting** to be:

1= Not at all important 5 = Very important

| [ ] | [ ] | [ ] | [ ] | [ ] |
| --- | --- | --- | --- | --- |
| 1 | 2 | 3 | 4 | 5 |

10.) Please use the scale below to rate how important you believe **accessible training opportunities for all staff regarding components of childhood resiliency building for all staff working within the pediatric medical home setting** to be:

1= Not at all important 5 = Very important

| [ ] | [ ] | [ ] | [ ] | [ ] |
| --- | --- | --- | --- | --- |
| 1 | 2 | 3 | 4 | 5 |

Practice:

11.) Please use the scale below to rate the likelihood that you incorporate **the central tenet of trauma informed care** into your routine patient visits:

1= Unlikely 5 = Very likely

| [ ] | [ ] | [ ] | [ ] | [ ] |
| --- | --- | --- | --- | --- |
| 1 | 2 | 3 | 4 | 5 |

12.) Please use the scale below to rate the likelihood that you incorporate **direct inquiry regarding traumatic exposures** into your routine patient visits:

1= Unlikely 5 = Very likely

| [ ] | [ ] | [ ] | [ ] | [ ] |
| --- | --- | --- | --- | --- |
| 1 | 2 | 3 | 4 | 5 |

13.) Please use the scale below to rate the likelihood that you incorporate **discussion with patients and caregivers regarding the relationship between history of traumatic exposures and poor health outcomes** into your routine patient visits:

1= Unlikely 5 = Very likely

| [ ] | [ ] | [ ] | [ ] | [ ] |
| --- | --- | --- | --- | --- |
| 1 | 2 | 3 | 4 | 5 |

14.) Please use the scale below to rate the likelihood that you incorporate **discussion of childhood resilience and resiliency building** into your routine patient visits:

1= Unlikely 5 = Very likely

| [ ] | [ ] | [ ] | [ ] | [ ] |
| --- | --- | --- | --- | --- |
| 1 | 2 | 3 | 4 | 5 |

Confidence**:**

15.) Please use the scale below to rate your confidence with **screening for childhood traumatic exposures**:

1= Not confident 5 = Very confident

| [ ] | [ ] | [ ] | [ ] | [ ] |
| --- | --- | --- | --- | --- |
| 1 | 2 | 3 | 4 | 5 |

16.) Please use the scale below to rate your confidence with **discussion with patients and caregivers regarding the relationship between history of traumatic exposures and poor health outcomes**:

1= Not confident 5 = Very confident

| [ ] | [ ] | [ ] | [ ] | [ ] |
| --- | --- | --- | --- | --- |
| 1 | 2 | 3 | 4 | 5 |

17.) Please use the scale below to rate your confidence with **discussion of resiliency building with patients and caregivers**:

1= Not confident 5 = Very confident

| [ ] | [ ] | [ ] | [ ] | [ ] |
| --- | --- | --- | --- | --- |
| 1 | 2 | 3 | 4 | 5 |

**POST TEST:**

Knowledge:

1.) Please use the scale to rate your general knowledge on **Adverse Childhood Experiences:**

1= Very Weak 5 = Very strong

| [ ] | [ ] | [ ] | [ ] | [ ] |
| --- | --- | --- | --- | --- |
| 1 | 2 | 3 | 4 | 5 |

2.) Please use the scale to rate your general knowledge on the **link between traumatic exposures and poor health outcomes:**

1= Very Weak 5 = Very strong

| [ ] | [ ] | [ ] | [ ] | [ ] |
| --- | --- | --- | --- | --- |
| 1 | 2 | 3 | 4 | 5 |

3.) Please use the scale below to rate your general knowledge on **the central tenet of trauma informed care:**

1= Very Weak 5 = Very strong

| [ ] | [ ] | [ ] | [ ] | [ ] |
| --- | --- | --- | --- | --- |
| 1 | 2 | 3 | 4 | 5 |

4.) Please use the scale below to rate your general knowledge on **the six principles of trauma informed care:**

1= Very Weak 5 = Very strong

| [ ] | [ ] | [ ] | [ ] | [ ] |
| --- | --- | --- | --- | --- |
| 1 | 2 | 3 | 4 | 5 |

5.) Please use the scale below to rate your general knowledge on **the components of childhood resilience:**

Attitudes:

6.) Please use the screen below to rate how important you **believe screening for traumatic exposures during routine patient visits in the pediatric medical home setting** to be:

1= Not at all important 5 = Very important

| [ ] | [ ] | [ ] | [ ] | [ ] |
| --- | --- | --- | --- | --- |
| 1 | 2 | 3 | 4 | 5 |

7.) Please use the screen below to rate how important you believe **implementation of clinic protocols to standardize screening for and addressing a history of traumatic exposures in patients and caregivers in the pediatric medical home setting** to be:

1= Not at all important 5 = Very important

| [ ] | [ ] | [ ] | [ ] | [ ] |
| --- | --- | --- | --- | --- |
| 1 | 2 | 3 | 4 | 5 |

8.) Please use the scale below to rate how important you believe **discussion with patients and caregivers regarding the relationship between a history of trauma exposure and poor health outcomes** to be:

1= Not at all important 5 = Very important

| [ ] | [ ] | [ ] | [ ] | [ ] |
| --- | --- | --- | --- | --- |
| 1 | 2 | 3 | 4 | 5 |

9.) Please use the scale below to rate how important you believe **accessible training opportunities regarding the relationship between trauma and health and the principles of trauma informed care for all staff working within the pediatric medical home setting** to be:

1= Not at all important 5 = Very important

| [ ] | [ ] | [ ] | [ ] | [ ] |
| --- | --- | --- | --- | --- |
| 1 | 2 | 3 | 4 | 5 |

10.) Please use the scale below to rate how important you believe **accessible training opportunities for all staff regarding components of childhood resiliency building for all staff working within the pediatric medical home setting** to be:

1= Not at all important 5 = Very important

| [ ] | [ ] | [ ] | [ ] | [ ] |
| --- | --- | --- | --- | --- |
| 1 | 2 | 3 | 4 | 5 |

Practice:

11.) Please use the scale below to rate the likelihood that you incorporate **the central tenet of trauma informed care** into your routine patient visits:

1= Unlikely 5 = Very likely

| [ ] | [ ] | [ ] | [ ] | [ ] |
| --- | --- | --- | --- | --- |
| 1 | 2 | 3 | 4 | 5 |

12.) Please use the scale below to rate the likelihood that you incorporate **direct inquiry regarding traumatic exposures** into your routine patient visits:

1= Unlikely 5 = Very likely

| [ ] | [ ] | [ ] | [ ] | [ ] |
| --- | --- | --- | --- | --- |
| 1 | 2 | 3 | 4 | 5 |

13.) Please use the scale below to rate the likelihood that you incorporate **discussion with patients and caregivers regarding the relationship between history of traumatic exposures and poor health outcomes** into your routine patient visits:

1= Unlikely 5 = Very likely

| [ ] | [ ] | [ ] | [ ] | [ ] |
| --- | --- | --- | --- | --- |
| 1 | 2 | 3 | 4 | 5 |

14.) Please use the scale below to rate the likelihood that you incorporate **discussion of childhood resilience and resiliency building** into your routine patient visits:

1= Unlikely 5 = Very likely

| [ ] | [ ] | [ ] | [ ] | [ ] |
| --- | --- | --- | --- | --- |
| 1 | 2 | 3 | 4 | 5 |

Confidence**:**

15.) Please use the scale below to rate your confidence with **screening for childhood traumatic exposures**:

1= Not confident 5 = Very confident

| [ ] | [ ] | [ ] | [ ] | [ ] |
| --- | --- | --- | --- | --- |
| 1 | 2 | 3 | 4 | 5 |

16.) Please use the scale below to rate your confidence with **discussion with patients and caregivers regarding the relationship between history of traumatic exposures and poor health outcomes**:

1= Not confident 5 = Very confident

| [ ] | [ ] | [ ] | [ ] | [ ] |
| --- | --- | --- | --- | --- |
| 1 | 2 | 3 | 4 | 5 |

17.) Please use the scale below to rate your confidence with **discussion of resiliency building with patients and caregivers**:

1= Not confident 5 = Very confident

| [ ] | [ ] | [ ] | [ ] | [ ] |
| --- | --- | --- | --- | --- |
| 1 | 2 | 3 | 4 | 5 |

_______________________________________________________________________

**Course Specific Feedback:**

1.) Please rate the quality of the module:

| [ ] | [ ] | [ ] | [ ] | [ ] |
| --- | --- | --- | --- | --- |
| 1 | 2 | 3 | 4 | 5 |
| Very Poor | Poor | Fair | Good | Very Good |

2.) Please provide any feedback or comments that you have on this course. Responses can relate to, but should not be limited to comments on (1) Organization, (2) Usability of learning interface (3) Course content (4) Applicability of information in clinical realm

3.) Please list 2 specific learning points that you will take away from this course:

4.) Please list 2 specific changes that you plan to make in your clinical practice as a result of this course:
